# A Role of Caveolae in Trabecular Meshwork Mechanosensing and Contractile Tone

**DOI:** 10.3389/fcell.2022.855097

**Published:** 2022-03-17

**Authors:** Michael L. De Ieso, Megan Kuhn, Pascal Bernatchez, Michael H. Elliott, W. Daniel Stamer

**Affiliations:** ^1^ Department of Ophthalmology, Duke Eye Center, Duke University, Durham, NC, United States; ^2^ Department of Anesthesiology, Pharmacology and Therapeutics, Faculty of Medicine, University of British Columbia, Heart + Lung Innovation Centre, St. Paul’s Hospital, Vancouver, BC, Canada; ^3^ Department of Ophthalmology, Dean McGee Eye Institute University of Oklahoma Health Sciences Center, Oklahoma City, OK, United States

**Keywords:** caveolae, trabecular meshwork, Rho/ROCK pathway, primary open angle glaucoma, contractility and relaxation, caveolin-1 (CAV1), caveolin-1 scaffolding domain peptide, cyclic stretch

## Abstract

Polymorphisms in the CAV1/2 gene loci impart increased risk for primary open-angle glaucoma (POAG). CAV1 encodes caveolin-1 (Cav1), which is required for biosynthesis of plasma membrane invaginations called caveolae. Cav1 knockout mice exhibit elevated intraocular pressure (IOP) and decreased outflow facility, but the mechanistic role of Cav1 in IOP homeostasis is unknown. We hypothesized that caveolae sequester/inhibit RhoA, to regulate trabecular meshwork (TM) mechanosensing and contractile tone. Using phosphorylated myosin light chain (pMLC) as a surrogate indicator for Rho/ROCK activity and contractile tone, we found that pMLC was elevated in Cav1-deficient TM cells compared to control (131 ± 10%, *n* = 10, *p* = 0.016). Elevation of pMLC levels following Cav1 knockdown occurred in cells on a soft surface (137 ± 7%, *n* = 24, *p* < 0.0001), but not on a hard surface (122 ± 17%, *n* = 12, *p* = 0.22). In Cav1-deficient TM cells where pMLC was elevated, Rho activity was also increased (123 ± 7%, *n* = 6, *p* = 0.017), suggesting activation of the Rho/ROCK pathway. Cyclic stretch reduced pMLC/MLC levels in TM cells (69 ± 7% *n* = 9, *p* = 0.002) and in Cav1-deficient TM cells, although not significantly (77 ± 11% *n* = 10, *p* = 0.059). Treatment with the Cav1 scaffolding domain mimetic, cavtratin (1 μM) caused a reduction in pMLC (70 ± 5% *n* = 7, *p* = 0.001), as did treatment with the scaffolding domain mutant cavnoxin (1 μM) (82 ± 7% *n* = 7, *p* = 0.04). Data suggest that caveolae differentially regulate RhoA signaling, and that caveolae participate in TM mechanotransduction. Cav1 regulation of these key TM functions provide evidence for underlying mechanisms linking polymorphisms in the Cav1/2 gene loci with increased POAG risk.

## Introduction

Ocular hypertension or elevated intraocular pressure (IOP) is the principal risk factor for glaucoma (Flammer and Mozaffarieh 2007), a leading cause of blindness worldwide (Tham et al., 2014). Ocular hypertension is also the only modifiable risk factor for primary open-angle glaucoma (POAG), and is due to the dysregulated resistance to drainage of aqueous humor from the eye *via* the conventional outflow (CO) pathway. The CO pathway consists of a porous connective tissue called the trabecular meshwork (TM), a specialized drainage vessel known as the Schlemm’s canal (SC), and distal drainage vessels that include collector channels, aqueous veins, and intrascleral venous plexus (Kizhatil et al., 2014). Some resistance to aqueous outflow is generated in the distal portion of the CO pathway (McDonnell et al., 2018), but the majority of outflow resistance is generated by the innermost region of the TM called the “juxtacanalicular (JCT)” region, where the inner wall of SC and the adjacent TM cells interact (Overby et al., 2009; Tamm 2009). Cells in the JCT region are mechanosensitive, and homeostatically respond to changes in IOP, thereby regulating resistance to aqueous humor outflow (Borras et al., 2002; Stamer et al., 2011; Acott et al., 2014; Overby et al., 2014; Stamer et al., 2015). The TM primarily participates in the regulation of outflow resistance by modulating extracellular matrix (ECM) turnover (Keller et al., 2009) and its contractile tone (Rao and Epstein 2007; Ren et al., 2016).

Since the JCT separates two pressure compartments (IOP versus episcleral vessel pressure), TM cells “sense” and respond to changes in IOP by alterations in mechanical strain. Such mechanical changes distort ion channels (Tran et al., 2014; Yarishkin et al., 2021), cell-cell and cell matrix junctional protein interactions (Tumminia et al., 1998; WuDunn 2009; Lakk and Križaj 2021) and caveolae (Elliott et al., 2016). Caveolae are specialized cellular domains that form “cup-shaped” invaginations in the plasma membrane (Richter et al., 2008; Schlormann et al., 2010), and are expressed abundantly in cells of the CO pathway including the SC and the TM (Tamm 2009; Herrnberger et al., 2012). Caveolae participate in a number of physiological processes including membrane trafficking (Parton et al., 1994; Pelkmans et al., 2001; Pelkmans and Helenius 2002), lipid and cholesterol regulation (Martin and Parton 2005; Pilch et al., 2007), cellular signaling pathways (Garcia-Cardena et al., 1996; Garcia-Cardena et al., 1997; Patel et al., 2008), mechanotransduction (Yu et al., 2006; Albinsson et al., 2008; Joshi et al., 2012), and mechanoprotection (Cheng et al., 2015; Lo et al., 2015). Caveolin-1 (Cav1) and caveolin-2 (Cav2) are protein scaffolds required for caveolae biosynthesis, and ablation of Cav1 results in loss of caveolae (Drab et al., 2001). Importantly, genetic association studies reproducibly show that polymorphisms at the Cav1/Cav2 gene loci increase risk for POAG and ocular hypertension (Thorleifsson et al., 2010; Wiggs et al., 2011; van Koolwijk et al., 2012; Chen et al., 2014; Huang et al., 2014; Hysi et al., 2014; Loomis et al., 2014; Ozel et al., 2014; Kim et al., 2015). In physiological studies, we previously found that global Cav1 knock out mice have elevated IOP due to decreased outflow facility (Elliott et al., 2016; Kizhatil et al., 2016; Lei et al., 2016), and that Cav1 expression in the TM is sufficient to rescue CO defects in global Cav1 knockout mice (De Ieso et al., 2020). Therefore, Cav1 expression in the TM appear essential for homeostatic regulation of IOP and CO, however, the biochemical and biomechanical mechanisms that underpin Cav1-facilitated regulation of CO resistance in the TM are still unknown.

Due to its central role in controlling TM contractility, the small GTPase, RhoA, is a prime candidate for caveolae-mediated transduction of changes in mechanical strain to homeostatic adjustments in TM contractile tone. RhoA switches between an inactive GDP-bound conformation and an active GTP-bound conformation, which triggers specific downstream effector proteins including Rho-associated protein kinase (ROCK) (Hall 2005). ROCK then phosphorylates and inactivates myosin light-chain phosphatase, leading to myosin light chain II phosphorylation and subsequent cross-linking of actin filaments and generation of contractile force (Hall 2005). Significantly, inhibition of Rho/ROCK signaling causes relaxation of the TM, resulting in reduced IOP and CO resistance (Rao and Epstein 2007; Ren et al., 2016). Cav1 physically interacts with RhoA in certain cell types (Gingras et al., 1998), likely *via* the Cav1 scaffolding domain (Okamoto et al., 1998; Taggart et al., 2000). Interestingly, Cav1 positively regulates Rho/ROCK activity and contractility in certain preparations (Grande-García et al., 2007; Peng et al., 2007; Joshi et al., 2008; Goetz et al., 2011; Yang et al., 2011; Parton and Del Pozo 2013; Echarri and Del Pozo 2015), and negatively regulates Rho/ROCK activity in other preparations (Gingras et al., 1998; Taggart et al., 2000; Nuno et al., 2012). TM cells possess phenotypic similarities to smooth muscle cells (Stamer and Clark 2017), where Cav1 has been reproducibly implicated in the negative regulation of Rho/ROCK activity and contractility (Taggart et al., 2000; Shakirova et al., 2006; Nuno et al., 2012). In addition, actin stress fiber formation (an effector of Rho/ROCK activity) is elevated in Cav1-deficient human TM cells (Aga et al., 2014).

Therefore, we hypothesized that caveolae sequester/inhibit RhoA, thus regulating downstream signaling involved in contractile tone and mechanosensing in the TM. To test this hypothesis, we employed adenoviruses encoding shRNA targeted to Cav1 to knock down its expression in primary cultures of human TM cell strains, and we used cell permeable Cav1 scaffolding domain-derived peptides to mimic elevation of Cav1 scaffolding domain activity. We observed that Cav1 deficiency resulted in elevated Rho/ROCK activity, and that treatment with Cav1 scaffolding domain peptides reduced Rho/ROCK activity. We also found that human TM cells downregulate Rho/ROCK signaling in response to cyclic mechanical stretch, and that this process is partially Cav1-dependent. Thus, work here reveals underlying mechanisms linking polymorphisms in the Cav1/2 gene loci with increased risk of POAG, and that the interaction between Cav1 and RhoA in the TM is important for mechanosensation and for the regulation CO resistance and function.

## Materials and Methods

### Human Trabecular Meshwork Cell Culture

Human TM cells were isolated from donor eyes using a blunt dissection technique followed by an extracellular matrix digestion protocol, exactly as previously described (Stamer et al., 1995). TM cells were characterized following established standards, including confirmation of proper TM cell morphology, and dexamethasone (100 nM for 5 days) induction of myocilin protein by immunofluorescence microscopy and western blot analysis according to consensus recommendations (Keller et al., 2018). Cells were cultured in low glucose Dulbecco’s modified Eagles’s medium (DMEM), containing 10% fetal bovine serum (FBS), 100 U/ml penicillin, 0.1 mg/ml streptomycin and maintained in humidified air containing 5% CO2 at 37°C. See Table 1 for a list of the TM cell strains used in this study, with available donor information.

**TABLE 1:** TM cell strains and eye donor information.

TM strain	Age	Sex	Race
hTM 89	64 y	?	?
hTM 96a	28 y	M	White
hTM 120	11 months	?	?
hTM 122	54 y	?	?
hTM 123	39 y	M	White
hTM 125	?	?	?
hTM 129	75 y	F	White
hTM 134	51 y	M	White
hTM 136	3 months	F	White
hTM 140	60 y	M	Black
hTM 144	75 y	F	White
hTM 150	4 months	F	Black
hTM 204	60 y	?	?
hTM 210	54 y	F	White
hTM 212	3 y	?	?

### Cell Transduction With Adenovirus

Cav1 expression in hTM cells was silenced using replication-deficient adenoviral constructs containing CAV1-silencing shRNAs and a GFP reporter (human adenovirus Type 5, dE1/E3 backbone, SKU# shADV-204148; Vector Biolabs, Malvern, PA). Control cells were transduced with particles containing the same vector encoding GFP and scrambled control shRNAs (Cat. #1122, Ad-GFP-U6-shRNA; Vector Biolabs). The optimal multiplicity of infection (MOI) for maximal Cav1 knockdown and minimal viral side effects was determined to be 10–15. We tested 1, 5, 10, 15, 20, and 50 MOI in the hTM120 cell strain to determine MOI that produced the greatest level of Cav1 knockdown with the least amount of observable undesirable effects, including change in cell morphology, and elevated expression of αSMA. As per manufacturer guidelines, cells were incubated in adenovirus solution (1 ml per well in 6-well plate; 2 ml per T25 flask; or 5 ml per T75 flask) for 6 h (5% CO_2_, 37°C), then topped up with fresh 10% FBS DMEM. Proteins were extracted at 72–96 h post viral transduction.

### Cyclic Mechanical Stretch of hTM Cells

Cells were seeded at confluence on 6-well Flexcell plates (Flexcell International Corp., Burlington, NC) that were coated with type IV collagen-coated as previously described (Hauser et al., 2015; Elliott et al., 2016; Youngblood et al., 2020). Cells were incubated in 1% FBS DMEM 2 h prior to starting cyclic stretch. *In vivo*, TM cells are bathed and nourished in aqueous humor, which contains about 1% serum proteins (Freddo 2013; Keller et al., 2018). Thus, in an attempt to simulate an *in vivo* environment, we chose to use 1% FBS instead of 0% FBS for use in experiments, which can unduly stress the cells. Cell stretch (20% stretch) was performed at frequency of 1 Hz, mimicking the ocular pulse, for 24 h in 1% FBS DMEM using the Flexcell FX-5000 (Flexcell International Corp., Burlington, NC). Evaluation of cells at 24-h was selected based on previous studies (Bradley et al., 2001; Bradley et al., 2003; Vittal et al., 2005).

### Western Blots

After 24 h of cyclic stretching, Flexcell plates were immediately placed on ice, cells were rinsed three times with ice-cold phosphate-buffered saline (PBS) and harvested by scraping into 2X Laemmli sample buffer with 1/10 dilution of beta mercaptoethanol (200μl/well of a 6-well plate), as performed previously (Dismuke et al., 2014). Protein lysates were boiled for 5 min and stored at −80°C. Equal volumes of protein lysate were loaded into 12% polyacrylamide gels, and proteins were separated by SDS-PAGE and transferred electrophoretically to nitrocellulose membranes using transfer buffer with 20% methanol. Blots were blocked at room temperature, on a rocker, for 60 min with 5% bovine serum albumin (BSA) in tris-buffered saline with 0.2% tween-20 (TBS-T). Primary antibodies were diluted in 5% BSA in TBS-T, and blots were incubated in primary antibody solution at 4°C overnight, on a rocker. The phosphorylation status of MLC was used as a surrogate indicator for Rho/ROCK activity and contractile tone as done previously (Kimura et al., 1996; Kureishi et al., 1997; Kaneko-Kawano et al., 2012). For analysis of MLC phosphorylation following Cav1 knockdown in TM cells plated on soft or hard surface, we combined data from multiple experiments that were completed for several different datasets, where cells were either plated on hard or soft surfaces. Therefore, some cells were treated with DMSO (0.05%) for 24 h prior to cell extraction, and others were not. Primary antibodies used are as follows: rabbit polyclonal anti-MLC (catalog number 3672; 1/1,000; Cell Signaling Technology), rabbit polyclonal anti-phospho-MLC (catalog number 3674; 1/1,000; Cell Signaling Technology), mouse monoclonal anti-alpha-tubulin HRP conjugate (catalog number 12351; 1/1,000; Cell Signaling Technology), rabbit monoclonal anti-caveolin-1 HRP conjugate (catalog number 12506; 1/1,000; Cell Signaling Technology), mouse monoclonal anti-phospho-caveolin-1 (catalog number 611338; 1/1,000; BD Biosciences), rabbit polyclonal anti-PTRF/cavin-1 (catalog number ABT131; 1/1,000; Millipore Sigma), mouse monoclonal anti-RhoA (catalog number sc-418; 1/200; Santa Cruz), and mouse monoclonal anti-alpha-smooth muscle actin (catalog number A2547; 1/5,000; Millipore Sigma). The blots were then washed three times (10 min in TBS-T). Membranes probed with non-HRP-conjugate antibodies were then incubated with goat anti-rabbit-HRP or anti-mouse-HRP secondary antibodies (1:5,000) for 1 h at room temperature, then washed three times (5 min in TBS-T). Blots were exposed using the ChemiDoc Imaging System (BioRad) and densitometry of protein bands was measured by Image Lab software (BioRad). It was not possible to run all samples on the same blot as there were not enough wells in the polyacrylamide gel to allow for all conditions for each sample. We addressed this issue in two ways. Firstly, all conditions for each cell strain or independent experiment were always probed on the same blot. Secondly, target protein band signal intensity was normalized to corresponding alpha-tubulin signal intensity on the same membrane, to correct for loading and variations in protein concentration for each respective sample. Additionally, pMLC and MLC were probed on separate blots due to inability to strip and reprobe for MLC proteins. For this reason, pMLC and MLC proteins were normalized to α-tubulin on respective gels to correct for loading, prior to pMLC normalization to MLC.

### Caveolin-1 Scaffolding Domain Peptides

We tested two Cav-1 scaffolding domain peptides and a vehicle control. Cavtratin (catalog number 219482; Millipore Sigma) is a Cav-1 scaffolding domain peptide (amino acids 82–101) fused at the N-terminus to the cell-permeable Antennapedia internalization sequence (43–58). Cavnoxin was synthesized by Elim Biosciences (Hayward, CA) is identical to cavtratin, although it contains the T90,91 and F92 substitutions to alanine, disrupting the inhibitory action of the scaffolding domain on eNOS (Bernatchez et al., 2005), along with N-terminus acetylation and C-terminus amidation for peptide stability. The Antennapedia internalization sequence alone was used as a peptide control. Cells were washed once with serum-free media prior to treatment. Peptides were diluted in serum-free media and cells were treated for 24 h prior to cell lysis. During the dose optimization study, we found that 1 μM of peptides was tolerated well by the cells, but 10 μM of peptides [as used previously (Bernatchez et al., 2011)] had adverse effects on readouts and cell morphology. For example, high doses of the peptides caused the cells to shrink and detach from the substratum.

### Rho Activation Assay

Human TM cells were grown to confluence in T-75 cell culture flasks in 10% FBS DMEM. Adenoviral transduction to silence Cav1 expression was performed 72–96 h prior to cell lysis. GFP signal was confirmed using fluorescence microscopy 48 h post-transduction. Cells were incubated in 1% FBS DMEM 2 h prior to cell lysis. The Active Rho Detection Kit (catalog number 8820; Cell Signaling) was used to perform the Rho activity assay. Cells were washed once with ice cold PBS and lysed with Lysis/Binding/Wash Buffer supplemented with 1 mM of phenylmethylsulfonyl fluoride (LBP). Cells were scraped and transferred to appropriate centrifuge tubes. Tubes were centrifuged at 16,000 XG for 15 min at 4°C, and supernatant was transferred to a new tube, while cell pellet was discard. Protein concentration was determined using the Pierce BCA Protein Assay Kit (catalog number 23225; Thermofisher Scientific). Lysate from each of the treatments (500 μg) was incubated with GST-Rhotekin-Rho binding domain-agarose slurry by gently rocking at 4°C for 1 h (as per the manufacturer’s instructions). Equal amounts of cell lysate protein from untreated cells were incubated either with GTPγS or with GDP, which served as positive and negative controls, respectively. The agarose beads were then washed three times with LBP and suspended in reducing sample buffer (2X Laemmli sample buffer with 200 mM dithiothreitol). The samples were vortexed and incubated at room temperature for 2 min. The samples were then centrifuged at 6000xG for 2 min, and the eluted samples were boiled for 5 min and stored at −80°C for future use. The GTP-bound and total Rho was detected by western blot analysis using Rho rabbit antibody (specific for RhoA, RhoB, and RhoC) supplied in Active Rho Detection Kit. Blots were exposed using the ChemiDoc Imaging System (BioRad) and densitometry of protein bands was measured by Image Lab software (BioRad). Signal obtained from refined protein lysate containing GTP-bound Rho was normalized to total Rho from crude protein lysate.

### Immunofluorescence Microscopy

Circular glass coverslips (12 mm diameter) were sterilized with 70% ethanol and inserted into wells of Greiner 24-well tissue culture treated plates (catalog number M8812; Millipore Sigma). Two days before fixation, cells were seeded at a density of 0.3 × 10^5^ cells per well, and incubated in 10% FBS DMEM overnight to allow the cells to adhere to the glass coverslips. Cell media was then replaced with 1% FBS DMEM and incubated overnight. Cells were then rinsed twice with ice cold PBS and fixed with 4% PFA for 30 min on ice. Cells were washed 3 times with TX solution (1/1,000 dilution of Triton X-100 in PBS) and blocked at room temperature for 60 min in blocking buffer (1/10 dilution of goat serum in TX-solution). Cells were then probed at 4°C overnight on a rocker with rabbit monoclonal anti-caveolin-1 primary antibody (catalog number 3267; 1/400; Cell Signaling Technology), diluted in blocking buffer. Cells were washed 3 times for 10 min with TX solution, and then probed with species-specific secondary antibody (Alexa Fluor 488; goat anti-rabbit; final concentration 3.75 μg/ml; catalog number 111–545–144; Jackson Immuno Research Labs), diluted in TX-solution for 2 h at room temperature on a rocker. Cells were then washed 3 times on a rocker for 10 min each, and the protocol was repeated with mouse monoclonal anti-RhoA (catalog number sc-418; 1/50; Santa Cruz) and respective species-specific secondary antibody (Alexa Fluor 647; goat anti-mouse; final concentration 3.75 μg/ml; catalog number 115-605-146; Jackson Immuno Research Labs). Cells were then incubated with DAPI (1 μg/ml diluted in PBS) for 30 min at room temperature. Cells were briefly rinsed once with PBS, and cover slips were mounted onto microscope slides using Immu-Mount (catalog number 9990402; Fisher Scientific). Images were captured using a Nikon A1R confocal laser scanning microscope with 4X air, 20X air objective lenses. For the signal emitted by DAPI, the excitation wavelength was 405 nm with emission filters set to detect wavelengths between 425 and 475 nm. For the signal emitted by Alexa Fluor 488, the excitation wavelength was 488 nm with emission filters set to detect wavelengths between 500 and 550 nm. For the signal emitted by Alexa Fluor 647, the excitation wavelength was 640 nm with emission filters set to detect wavelengths between 663 and 738 nm. Fiji (ImageJ) software (United States National Institutes of Health) was used to identify subcellular colocalization of Cav1 and RhoA, by measuring signal intensities as a function of cross-sectional distance per image, as previously described (De Ieso et al., 2019; Pei et al., 2019). Unidimensional coincident peaks from Cav1 and RhoA signal were interpreted as colocalization. The inclusion criteria for selecting cross sections for analysis were as follows: 1) to be considered a peak, the maximal Y-value of the peak must have been greater than 10 units. 2) For each single image depicting a specific cell line, three cross-sectional lines were drawn. 3) Each line needed to pass through at least three cells.

### Subcellular Fractionation

At 96 h post viral transduction (as described above) cells were washed 3 times with ice-cold PBS and harvested by scraping into detergent-free buffer (PBS with 0.5 mM ethylenediaminetetraacetic acid, catalog number 03690; Millipore Sigma, Halt Protease Inhibitor Cocktail, catalog number 78425; ThermoFisher Scientific, and Phosphatase Inhibitor Cocktail 2, catalog number P5726; Millipore Sigma). Cell lysate was homogenized by drawing lysate up and down 10 times with a 27-gauge needle. Lysates were then gently centrifuged (800 XG) for 5 min to pellet nuclei and large debris. The supernatant (cytosol + membrane fractions) was removed and centrifuged at 16,000 XG for 15 min to separate the cytosol and membrane fractions. Detergent-free buffer (50 μl) was added to the nuclear fraction, which was then stored at −80°C. After the cytosolic and membrane fractions were separated, the supernatant (cytosol-enriched fraction) was removed and stored at −80°C. The membrane-enriched pellet was washed twice with detergent-free buffer and centrifuged again at 16,000 XG for 15 min. The supernatant was removed and discarded, and the pellet was solubilized in a buffer containing 1% Triton X-100 (40 μl) to lyse the membrane pellet. The membrane fraction was also stored at −80°C until protein quantification. Protein concentration was determined using the Pierce BCA Protein Assay Kit (catalog number 23225; Thermofisher Scientific). Protein lysates were mixed with reducing sample buffer (4X Laemmli sample buffer with 1/10 dilution of beta mercaptoethanol) and boiled for 5 min. Equal volumes of protein lysate (10 μg per well) were loaded into TGX Stain-Free FastCast 12% polyacrylamide gels (catalog number 1610184; Biorad). Proteins were separated by SDS-PAGE and gels were activated prior to being transferred electrophoretically to nitrocellulose membranes using transfer buffer with 20% methanol. Protein expression was corrected for loading via normalization to total protein. Alpha-tubulin was used as a cytosolic marker protein and Cav1 was used as a membrane marker protein to confirm purity of membrane and cytosolic fractions.

### Statistical Analysis

Graphing and statistical analyses were performed using GraphPad Prism v.9.2.0 (GraphPad Software, La Jolla, CA). A *p* value of 0.05 or less was considered significant and data are presented as mean ± SEM, unless otherwise stated. Significance was determined using paired and unpaired t-tests. We used unpaired t-tests when comparing the means of two independent or unrelated groups, for example, basal pMLC levels across different cell lines. Alternatively, we used a paired *t*-test when comparing means of the same group or item (the experiment/cell line) under two separate scenarios (e.g., stretch verses no stretch). This is to account for variability between cell strains and experiments, and a paired *t*-test is applicable here because within any given experiment, the cell strains were under identical conditions until the treatments (stretch or virus) were applied. Outliers were defined as values that lie two standard deviations outside of the mean.

## Results

### Cav1 Knockdown Increases Phosphorylation of Myosin Light Chain in Human TM Cells

**FIGURE 1:**
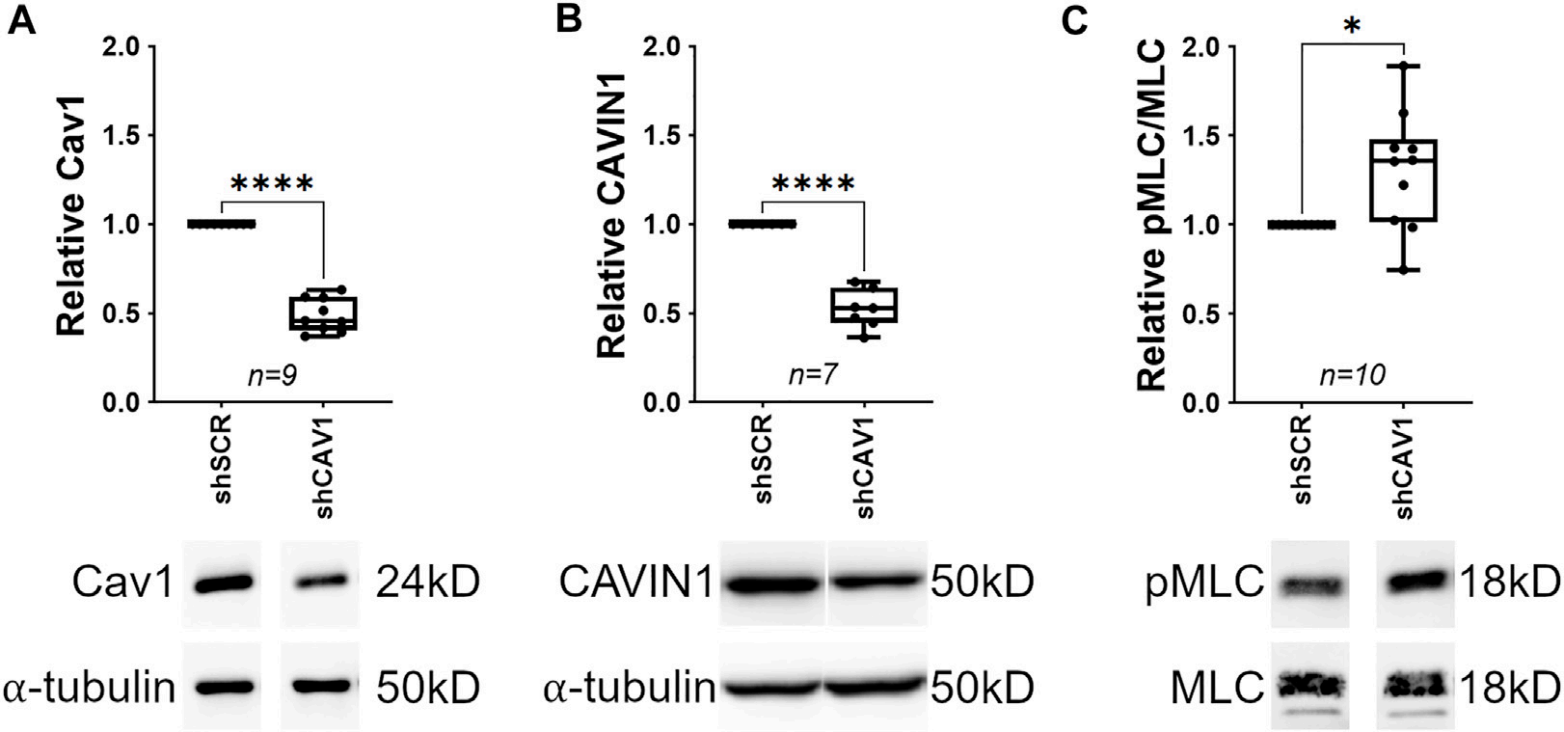
Cav1 knockdown increases phosphorylation of myosin light chain in human TM cells. (**(A)**, Top) Cav1 protein levels were significantly decreased by approximately 50% in TM cells treated with CAV1-silencing shRNA compared to cells treated with scrambled shRNA (****p < 0.0001, paired *t*-test, *n* = 9 experiments using six different human TM cell strains). (**(A)**, Bottom) Shown is a representative blot from a single experiment displaying Cav1 protein abundance, compared to α-tubulin. (**(B)**, Top) CAVIN1 protein levels were significantly decreased by approximately 50% in TM cells treated with CAV1-silencing shRNA compared to cells treated with scrambled shRNA (****p < 0.0001, paired *t*-test, *n* = 7, four different human TM cell strains tested). (**(B)**, Bottom) Shown is a representative blot for CAVIN1 protein, compared to α-tubulin. (**(C)**, Top) MLC phosphorylation was significantly elevated by approximately 30% in Cav1-deficient TM cells compared to Cav1-competent TM cells (*p = 0.016, paired *t*-test, *n* = 10 experiments using six different human TM cell strains). (**(C)**, Bottom) Representative blots depicting pMLC signal, compared to total MLC from a hTM cell strain. Summary data are displayed as box and whisker plots, which show the minimum, 25th percentile, median, 75th percentile, and maximum values of the dataset, plus all individual data points (black circles). Data are expressed as candidate protein expression (corrected for loading as indicated by α-tubulin abundance) in cells treated with shCAV1, normalized to shSCR treated cells.

Actin stress fiber formation (an effector of Rho/ROCK activity) is elevated in human TM cells depleted of Cav1 (Aga et al., 2014), and smooth muscle contractility is elevated in the absence of Cav1 in several other cell types (Shakirova et al., 2006). Therefore, we hypothesized that Cav1 depletion in the TM results in an elevation of Rho/ROCK activity and subsequently contractile tone. To test our hypothesis, we silenced Cav1 in human TM cells and monitored the phosphorylation status of myosin light chain (MLC), a surrogate indicator for Rho/ROCK activity and contractile tone (Kimura et al., 1996; Kureishi et al., 1997; Kaneko-Kawano et al., 2012). Results show a reproducible reduction of Cav1 in TM cells by over 50% using CAV1-silencing shRNAs compared to scrambled shRNAs (Figure 1A, 0.49 ± 0.032, *n* = 9, *p* < 0.0001). We also monitored a second indicator of Cav1 knockdown, CAVIN1, a Cav1 interacting transcription factor (Hill et al., 2008; Liu and Pilch 2008; Hansen et al., 2013). Accordingly, we observed that CAVIN1 was significantly reduced to similar levels as Cav1 in Cav1-deficient TM cells compared to control (Figure 1B, 0.52 ± 0.041, n = 7, *p* < 0.0001). In contrast, Cav1 knockdown had no effect on expression levels of αSMA, RhoA, or MLC (Supplementary Figure S1). In terms of MLC activation, we found that phosphorylated MLC was significantly elevated in Cav1-deficient TM cells compared to Cav1-competent TM cells (Figure 1C, 1.31 ± 0.1, *n* = 10, *p* = 0.016), suggesting Cav1 negatively regulates Rho/ROCK activity, and subsequently contractile tone in human TM cells.

### Elevation of MLC Phosphorylation Following Cav1 Knockdown is Cell Substrate-Dependent

**FIGURE 2:**
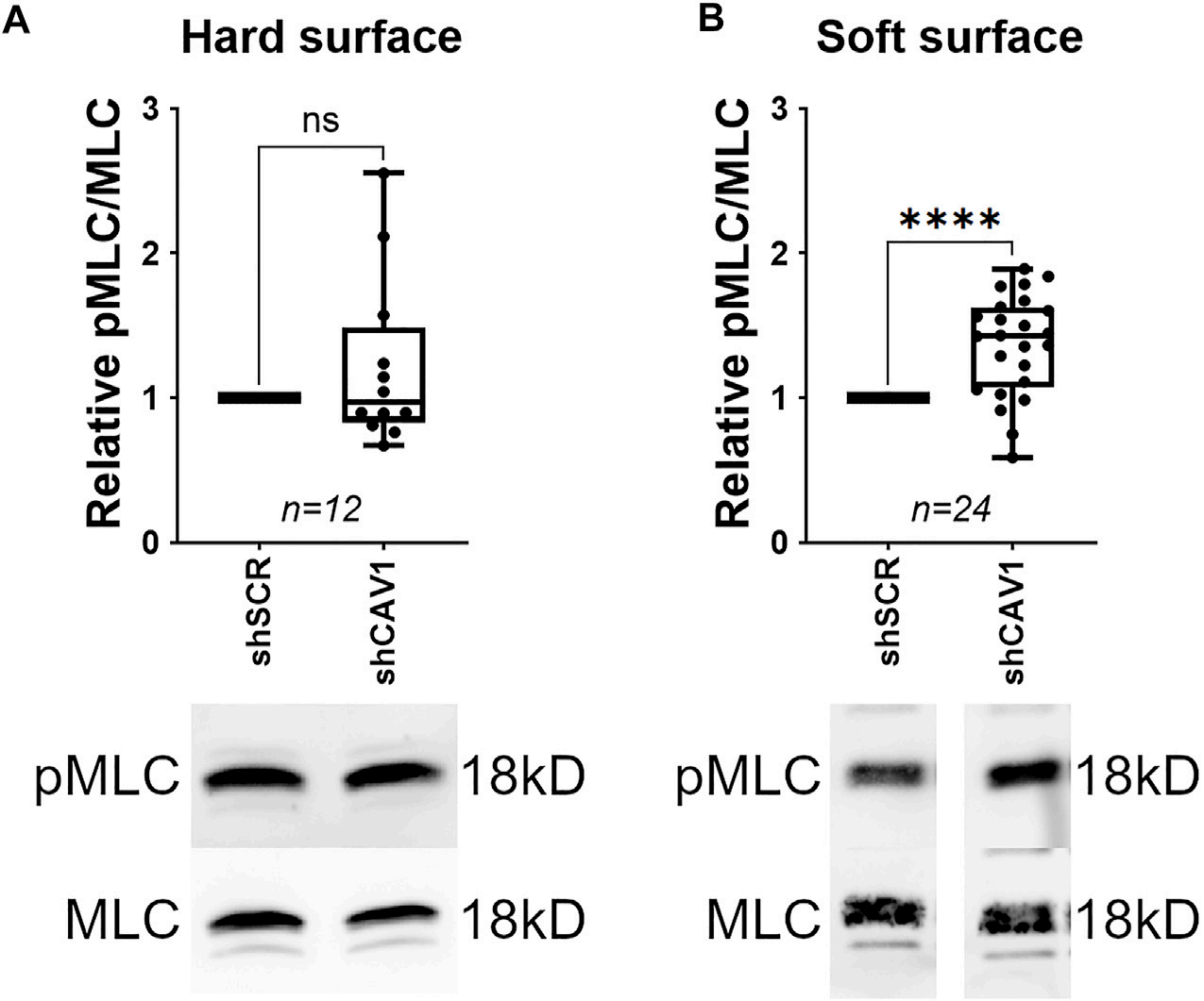
Elevation of MLC phosphorylation following Cav1 knockdown in TM cells on soft versus hard surface. (**(A)**, Top) MLC phosphorylation was not significantly different between Cav1-deficient and Cav1-competent TM cells when plated on a hard surface (*p* = 0.224, paired *t*-test, *n* = 12 experiments using seven different human TM cell strains). (**(A)**, Bottom) Shown is a representative blot from a single experiment displaying phosphorylated MLC abundance, compared to total MLC protein. (**(B)**, Top) In contrast, MLC phosphorylation was significantly elevated by approximately 37% in Cav1-deficient TM cells compared to Cav1-competent TM cells when plated on a soft surface (*p < 0.0001, paired *t*-test, *n* = 24 experiments using 11 different human TM cell strains). (**(B)**, Bottom) Representative blots from a single experiment displaying phosphorylated MLC abundance, compared to total MLC protein. Summary data are displayed as box and whisker plots, which show the minimum, 25th percentile, median, 75th percentile, and maximum values of the dataset, plus all individual data points (black circles). Data are expressed as candidate protein expression (corrected for loading as indicated by α-tubulin abundance) in cells treated with shCAV1, normalized to shSCR treated cells.

TM cells cultured on rigid substrates results in transcriptomic modifications for genes involved in ECM regulation, mechanotransduction, and glaucoma pathogenesis (Thomasy et al., 2012; Tie et al., 2020). Additionally, downstream effectors of Rho/ROCK signaling, such as stress fiber formation, are elevated in TM cells cultured on rigid substrates (Schlunck et al., 2008). Therefore, we hypothesized that the elevation of pMLC in response to Cav1 knockdown would be less pronounced when cells were plated on a hard surface rather than a soft surface. To test this hypothesis, we compared the effect of Cav1 knockdown between TM cells plated on flexible silicone bottom plates (soft) verses plastic (hard). As such, phosphorylated MLC was significantly elevated in Cav1-deficient TM cells compared to Cav1-competent TM cells when plated on a soft surface (Figure 2B, 1.37 ± 0.07, *n* = 24, *p* < 0.0001). In contrast, phosphorylated MLC was slightly elevated in Cav1-deficient TM cells compared to Cav1-competent TM cells, although not significantly for TM cells plated on hard surfaces, (Figure 2A, 1.22 ± 0.17, n = 12, *p* = 0.224). These data suggest that substrate stiffness influences Cav1-facilitated regulation of Rho/ROCK activity in human TM cells.

### Cav1 Knockdown Elevates MLC Phosphorylation *via* RhoA Activation in Human TM Cells

**FIGURE 3:**
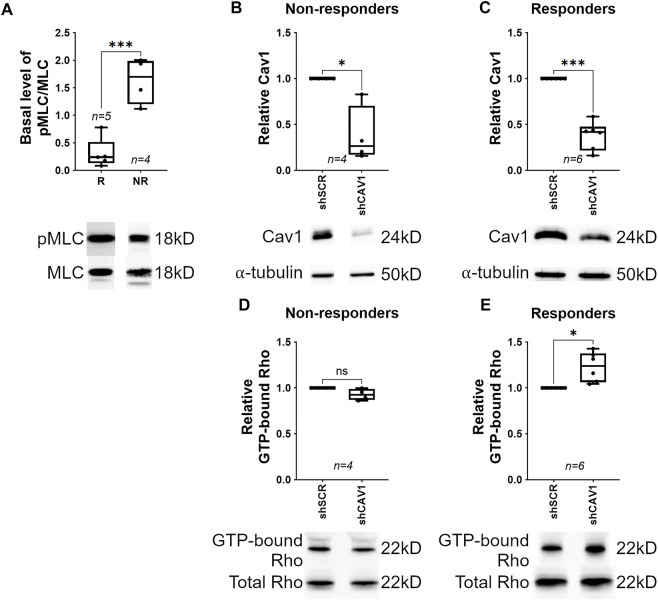
Cav1-knockdown elevates MLC phosphorylation via Rho activation in human TM cells. Responders (R) are defined as TM cells where MLC phosphorylation levels were elevated following Cav1 knockdown. Non-responders (NR) are defined as TM cells where MLC phosphorylation levels were not elevated following Cav1 knockdown. (**(A)**, Top) Basal MLC phosphorylation was significantly higher in NR (*n* = 4 experiments using four different human TM cell strains) compared to responders (R, *n* = 5 experiments using four different human TM cell strains) (***p = 0.0007, unpaired *t*-test). (**(A)**, Bottom) Shown is a representative blot from a single experiment displaying phosphorylated MLC abundance, compared to total MLC protein. (**(B)**, Top) In non-responders, Cav1 protein levels were significantly decreased by approximately 60% in TM cells treated with CAV1-silencing shRNA compared to cells treated with scrambled shRNA (*p = 0.027, paired *t*-test, *n* = 4 experiments using four different human TM cell strains). (**(B)**, Bottom) Shown is a representative blot from a single experiment displaying Cav1 protein abundance, compared to α-tubulin. (**(C)**, Top) In responders, Cav1 protein levels were comparable to non-responders being decreased by approximately 60% in TM cells treated with CAV1-silencing shRNA compared to cells treated with scrambled shRNA (***p = 0.0002, paired *t*-test, *n* = 6 experiments using four different human TM cell strains). (**(C)**, Bottom) Shown is a representative blot from a single experiment displaying Cav1 protein abundance, compared to α-tubulin. (**(D)**, Top) In non-responders, levels of GTP-bound Rho were not significantly different between Cav1-competent and Cav1-deficient TM cells (*p* = 0.097, paired *t*-test analysis, *n* = 4 experiments using four different human TM cell strains). (**(D)**, Bottom) Shown is a representative blot from a single experiment displaying GTP-bound Rho signal, normalized to total Rho protein. (**(E)**, Top) In contrast, GTP-bound Rho in responders was significantly elevated by approximately 23% in Cav1-deficient compared to Cav1-competent TM cells. (*p* = 0.017, paired *t*-test, *n* = 6 experiments using four different human TM cell strains). (**(E)**, Bottom) Shown is a representative blot from a single experiment displaying GTP-bound Rho signal, normalized to total Rho protein. Summary data are displayed as box and whisker plots, which show the minimum, 25th percentile, median, 75th percentile, and maximum values of the dataset, plus all individual data points (black circles). In A-C, data are expressed as candidate protein expression, corrected for loading as indicated by α-tubulin abundance. For D and E, expression of GTP-bound Rho is corrected for loading via normalization to total Rho, and not α-tubulin. For B-E, data are expressed as candidate protein expression in cells treated with shCAV1, normalized to shSCR treated cells.

We observed that the dampened response by TM cells plated on stiff surfaces could be segregation into two groups; “responders” having elevated pMLC and “non-responders” having low levels of pMLC with Cav1 knockdown. We hypothesized that basal (shSCR-treated) pMLC levels are high in non-responders, which would result in a “ceiling effect”; meaning further elevation of pMLC in response to Cav1 knockdown would be less pronounced. As depicted in Figure 3A, we discovered that in shSCR-treated TM cells, basal pMLC levels were more than 80% lower in responders (0.31 ± 0.12, *n* = 5) compared to non-responders (1.63 ± 0.21, *n* = 4, *p* = 0.0007). This suggested that elevation of pMLC in response to Cav1-deficiency primarily occurs in hTM cells where basal pMLC levels are not already elevated. As before, we reproducibly and equivalently reduced Cav1 in TM cells with CAV1-silencing shRNAs compared to scrambled shRNAs in non-responders (Figure 3B, 0.38 ± 0.15, *n* = 4, *p* = 0.027) and responders (Figure 3C, 0.38 ± 0.06, *n* = 6, *p* = 0.0002) and assayed for Rho activity. We observed that within any given experiment, changes in pMLC levels were directly proportional to changes in Rho activity following Cav1 knockdown. As such, in non-responders, Rho activity did not significantly change in Cav1-deficient compared to Cav1-competent TM cells (Figure 3D, 0.927 ± 0.03, *n* = 4, *p* = 0.097). In contrast, Rho activity in responders was significantly elevated in Cav1-deficient compared to Cav1-competent TM cells (Figure 3E, 1.23 ± 0.07, *n* = 6, *p* = 0.017), suggesting that Cav1-knockdown elevates MLC phosphorylation *via* Rho/ROCK pathway. As positive and negative controls for the Rho activity assays, we treated cell lysates with GTPγS or GDP, respectively (Supplementary Figure S2).

### Cav1 Scaffolding Domain Peptides Cause MLC Dephosphorylation in Human TM Cells

**FIGURE 4:**
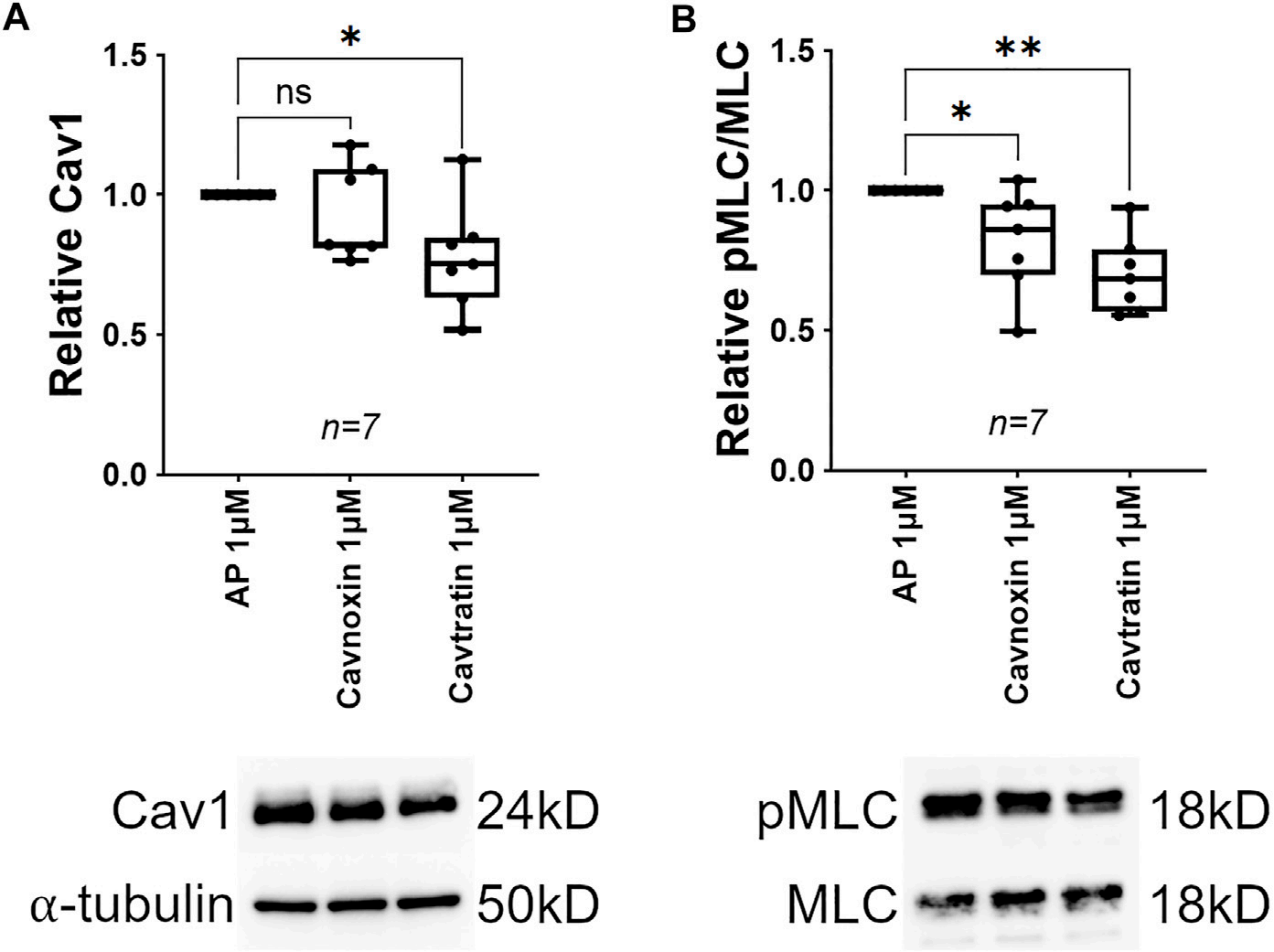
Cav1 scaffolding domain mimetics cause MLC dephosphorylation in human TM cells. (**(A)**, Top) Treatment with the peptide mimetic, cavnoxin (1 μM) for 24 h had no effect on Cav1 protein levels compared to 1 μM peptide control (Antennapedia internalization sequence, AP) (*p* = 0.333, paired *t*-test, *n* = 7 experiments using three different human TM cell strains). Treatment with the peptide mimetic, cavtratin (1 μM) for 24 h significantly reduced Cav1 protein levels by 22% compared to 1 μM AP (*p = 0.021, paired *t*-test, *n* = 7 experiments using three different human TM cell strains). (**(A)**, Bottom) Shown is a representative blot from a single experiment displaying Cav1 protein abundance, compared to α-tubulin. (**(B)**, Top) Treatment with 1 μM cavnoxin for 24 h significantly reduced MLC phosphorylation by 18% compared to 1 μM AP (*p = 0.046, paired *t*-test, *n* = 7 experiments using three different human TM cell strains). Treatment with 1 μM cavtratin for 24 h significantly reduced MLC phosphorylation by 30% compared to 1 μM AP (**p = 0.001, paired *t*-test, *n* = 7 experiments using three different human TM cell strains). (**(B)**, Bottom) Representative blots from a hTM cell strain depicting pMLC signal, normalized to total MLC. Summary data are displayed as box and whisker plots, which show the minimum, 25th percentile, median, 75th percentile, and maximum values of the dataset, plus all individual data points (black circles). Data are expressed as candidate protein expression (corrected for loading as indicated by α-tubulin abundance) in cells treated with cavtratin or cavnoxin, normalized to AP-treated cells.

The Cav1 scaffolding domain has been shown to inhibit signal transduction *via* sequestration of a number of key signaling molecules, including RhoA (Oka et al., 1997; Engelman et al., 1998; Razani et al., 1999; Bucci et al., 2000; Razani and Lisanti 2001; Gratton et al., 2003; Labrecque et al., 2003; Lu et al., 2018). For example, Cav1 physically interacts with, and inhibits RhoA in several cell types (Gingras et al., 1998), likely via the scaffolding domain (Okamoto et al., 1998; Taggart et al., 2000). Thus, as MLC phosphorylation is elevated following Cav1 knockdown, we hypothesized that treatment of TM cells with the Cav1 scaffolding domain peptides would mimic upregulation of Cav1 activation and cause MLC dephosphorylation. Interestingly, we found that treatment of TM cells with cavtratin for 24 h resulted in a 22% reduction in relative Cav1 protein as compared to control (Figure 4A, 0.775 ± 0.07, *n* = 7, *p* = 0.021). In contrast, treatment with cavnoxin had no significant effect on relative Cav1 protein levels as compared to control (Figure 4A, 0.933 ± 0.06, *n* = 7, *p* = 0.332). These data suggest that treatment with cavtratin initiated a negative feedback loop, causing a downregulation of Cav1 protein expression. In addition to decreased Cav1 expression, TM cells treated with cavtratin exhibited a 30% reduction in MLC phosphorylation as compared to control (Figure 4B, 0.699 ± 0.05, *n* = 7, *p* = 0.001). Cavnoxin was less efficacious than cavtratin, exhibiting an 18% reduction in MLC phosphorylation in TM cells as compared to AP control (Figure 4B, 0.82 ± 0.07, *n* = 7, *p* = 0.043). These data support the hypothesis that Cav1 negatively regulates Rho/ROCK signaling in human TM cells, *via* the Cav1 scaffolding domain.

### Cyclic Stretch Induces MLC Dephosphorylation in Cav1-Competent, not Cav1-Deficient TM Cells

**FIGURE 5:**
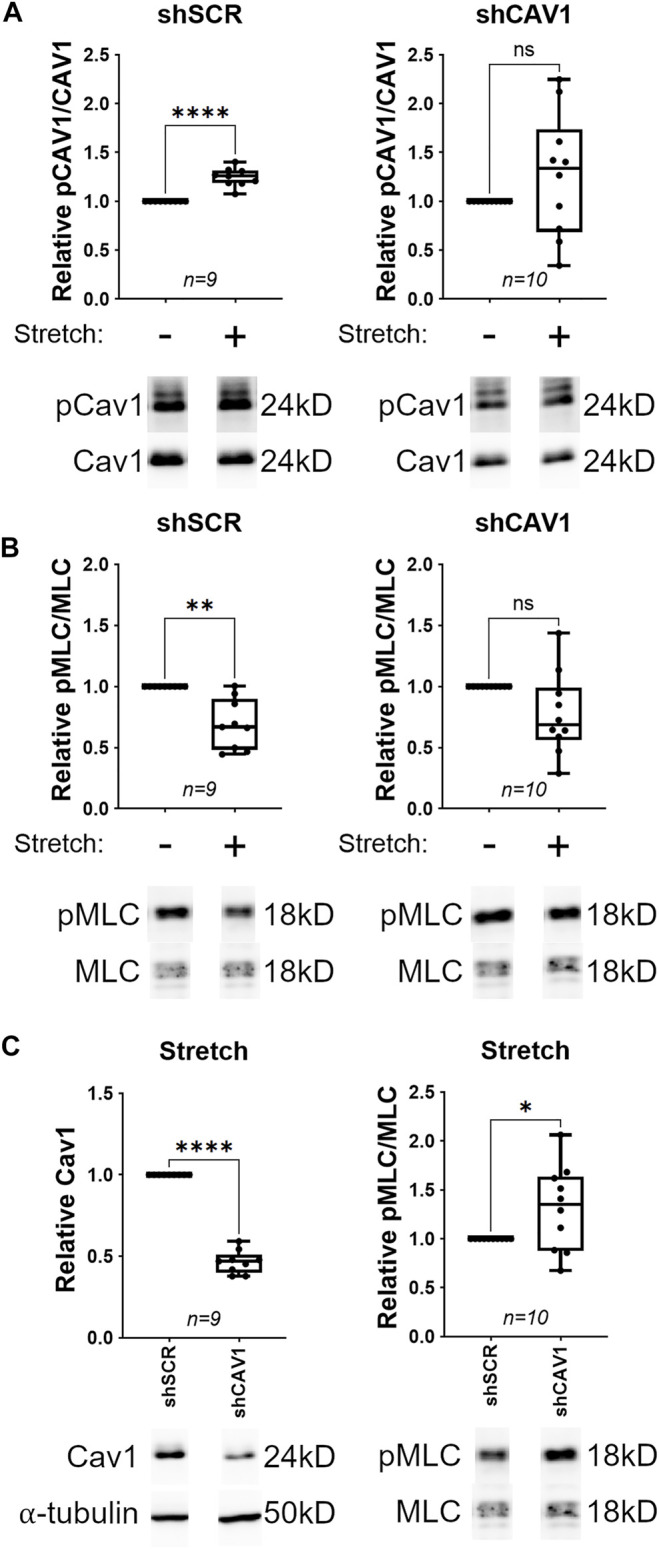
Cyclic stretch results in MLC dephosphorylation in Cav1-competent, but not Cav1-deficient TM cells. (**(A)**, Top) For Cav1-competent TM cells (treated with shSCR), cyclic stretch caused a 25% increase in Cav1 phosphorylation compared to unstretched TM cells (****p < 0.0001, paired *t*-test, *n* = 9 experiments using six different human TM cell strains). For Cav1-deficient TM cells (treated with shCAV1), cyclic stretch had no significant effect on Cav1 phosphorylation compared to unstretched TM cells (*p* = 0.213, paired *t*-test, *n* = 10 experiments using six different human TM cell strains). (**(A)**, Bottom) Representative blots from a hTM cell strain depicting pCAV1 signal, normalized to total CAV1. (**(B)**, Top) For Cav1-competent TM cells, cyclic stretch caused a 30% decrease in MLC phosphorylation compared to unstretched TM cells (**p = 0.002, paired *t*-test, *n* = 9 experiments using five different human TM cell strains). For Cav1-deficient TM cells, cyclic stretch had no significant effect on MLC phosphorylation compared to unstretched TM cells (*p* = 0.059, paired *t*-test, *n* = 10 experiments using six different human TM cell strains). (**(B)**, Bottom) Representative blots from a hTM cell strain depicting pMLC signal, normalized to total MLC. (**(C)**, Top Left) In stretched TM cells, Cav1 protein levels were significantly decreased by approximately 53% in cells treated with CAV1-silencing shRNA compared to cells treated with scrambled shRNA (****p < 0.0001, paired *t*-test, *n* = 9 experiments using six different human TM cell strains). (**(C)**, Bottom Left) Shown is a representative blot from a single experiment displaying Cav1 protein abundance, compared to α-tubulin. (**(C)**, Top Right) In stretched TM cells, Cav1-deficient cells exhibited a 30% elevation in MLC phosphorylation, as compared to Cav1-competent cells (*p = 0.049, paired *t*-test, *n* = 10 experiments using six different human TM cell strains). (**(C)**, Bottom Right) Representative blots from a hTM cell strain depicting pMLC signal, normalized to total MLC. Summary data are displayed as box and whisker plots, which show the minimum, 25th percentile, median, 75th percentile, and maximum values of the dataset, plus all individual data points (black circles). For A and B, data are expressed as candidate protein expression (corrected for loading as indicated by α-tubulin abundance) in stretched cells, normalized to unstretched cells. For C, data are expressed as candidate protein expression (corrected for loading as indicated by α-tubulin abundance) in shCAV1 treated cells, normalized to shSCR treated cells.

Caveolae are mechanotransducers in some tissues (Yu et al., 2006). For example, mechanical stimulation induces phosphorylation of Cav1 in breast cancer cells (Joshi et al., 2012). Since TM cells are subject to continual cyclic stretch (Xin et al., 2018), we aimed to determine whether cyclic mechanical stretch induces Cav1 phosphorylation in human TM cells. In Cav1-competent TM cells, 24 h of cyclic stretch caused a significant increase in Cav1 phosphorylation as compared to no stretch (Figure 5A, 1.25 ± 0.03, *n* = 9, *p* < 0.0001). In Cav1-deficient TM cells, 24 h of cyclic stretch elevated phosphorylated Cav1 levels to similar levels, but not significantly (Figure 5A, 1.27 ± 0.2, *n* = 10, *p* = 0.213). These results show that cyclic mechanical stretch stimulates phosphorylation of Cav1 in Cav1-competent TM cells, and that this effect is disrupted in the TM cells with lower levels of Cav1.

The ability of the conventional outflow tissues to adapt to fluctuating biomechanical loads is crucial to IOP homeostasis (Overby et al., 2014; Stamer et al., 2015). As MLC dephosphorylation and subsequent TM relaxation elevates outflow facility and reduces IOP (Rao et al., 2001; Rao et al., 2005; Ren et al., 2016), we hypothesized that TM cells respond to cyclic mechanical stretch by dephosphorylating MLC, and subsequently relaxing. Indeed, 24 h of cyclic stretch caused a significant decrease in MLC phosphorylation as compared to no stretch in Cav1-competent TM cells (Figure 5B, 0.693 ± 0.07, *n* = 9, *p* = 0.002). One hour of cyclic stretch was not sufficient to cause MLC dephosphorylation in Cav1-competent TM cells (Supplementary Figure S4). We then tried to determine whether the stretch-induced relaxation was Cav1 dependent. We observed that the response was more variable and did not reach significance in Cav1-deficient TM cells, whereby 24 h of cyclic stretch caused a 23% decrease in MLC phosphorylation as compared to no stretch (Figure 5B, 0.772 ± 0.11, *n* = 10, *p* = 0.059). These data suggest that cyclic mechanical stretch stimulates dephosphorylation of MLC in Cav1-competent TM cells, and that this effect is disrupted in the TM cells with lower levels of Cav1. We then only looked at TM cells that had been stretched for 24 h, and within this group we found that Cav1 was reduced by 53% with CAV1-silencing shRNAs compared to scrambled shRNAs (Figure 5C, 0.465 ± 0.02, *n* = 9, *p* < 0.0001). Moreover, phosphorylated MLC was significantly elevated by 31% in Cav1-deficient compared to Cav1 competent TM cells (Figure 5C, 1.31 ± 0.14, *n* = 10, *p* = 0.049). Importantly, we observed that 24 h of cyclic stretch had no effect on protein expression of Cav1, CAVIN1, αSMA, MLC, or RhoA (Supplementary Figure S3). These data suggest that in the presence or absence of cyclic mechanical stretch, MLC phosphorylation is elevated in Cav1-deficient TM cells.

### RhoA Co-Localizes With Cav1 in Human TM Cells

**FIGURE 6:**
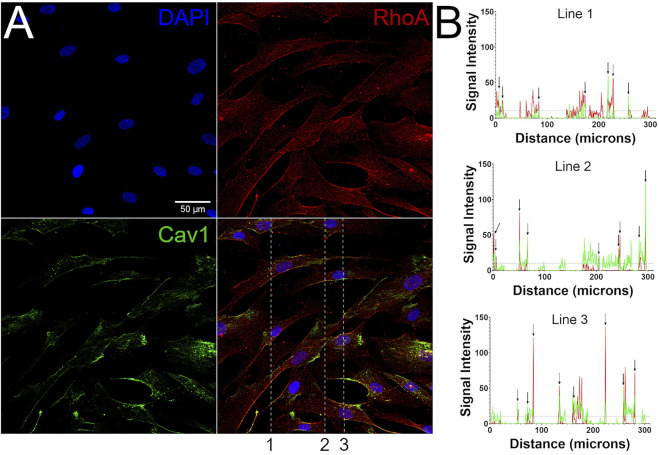
RhoA co-localizes with Cav1. **(A)** Immunofluorescence microscopic images depicting human TM cells, stained for DAPI (blue, top left), RhoA (red, top right), and Cav1 (green, bottom left). Merged signals are displayed on bottom right, with dotted lines showing locations where signal intensities were analyzed in the *Z*-axis in panel B. **(B)** Each graph shows the signal intensities (*y*-axis) of the Cav1 (green) and RhoA signals (red), as a function of distance (*x*-axis) along the dotted lines shown in merged panel A. Points of coincidental signal peaks (peak defined as maximum Y value > 10 arbitrary units) are representative of Cav1/RhoA co-localization and are highlighted with arrows. Shown are representative results from a single hTM cell strain (hTM150) of three total cell strains that were analyzed.

Cav1 physically interacts with Rho GTPases in non-TM cell types, likely via its scaffolding domain (Gingras et al., 1998; Okamoto et al., 1998; Taggart et al., 2000; Peng et al., 2007; Peng et al., 2008; Arpaia et al., 2012). These data plus our results showing that Cav1 regulates RhoA activity in TM cells, led us to hypothesize that Cav1 colocalizes with RhoA in human TM cells. Using immunofluorescent microscopy, we observed that the majority of Cav1 is expressed on the plasma membrane of TM cells. Similarly, we found that RhoA expression is primarily concentrated at cell borders, with some distributed throughout the cytoplasm (Figure 6A and Supplementary Figure S5A). Colocalization was confirmed in confocal images analyzed in cross sections (Figure 6B and Supplementary Figure S5B).

### RhoA Expression in the Cytosol is Suppressed in Cav1-Deficient TM Cells

**FIGURE 7:**
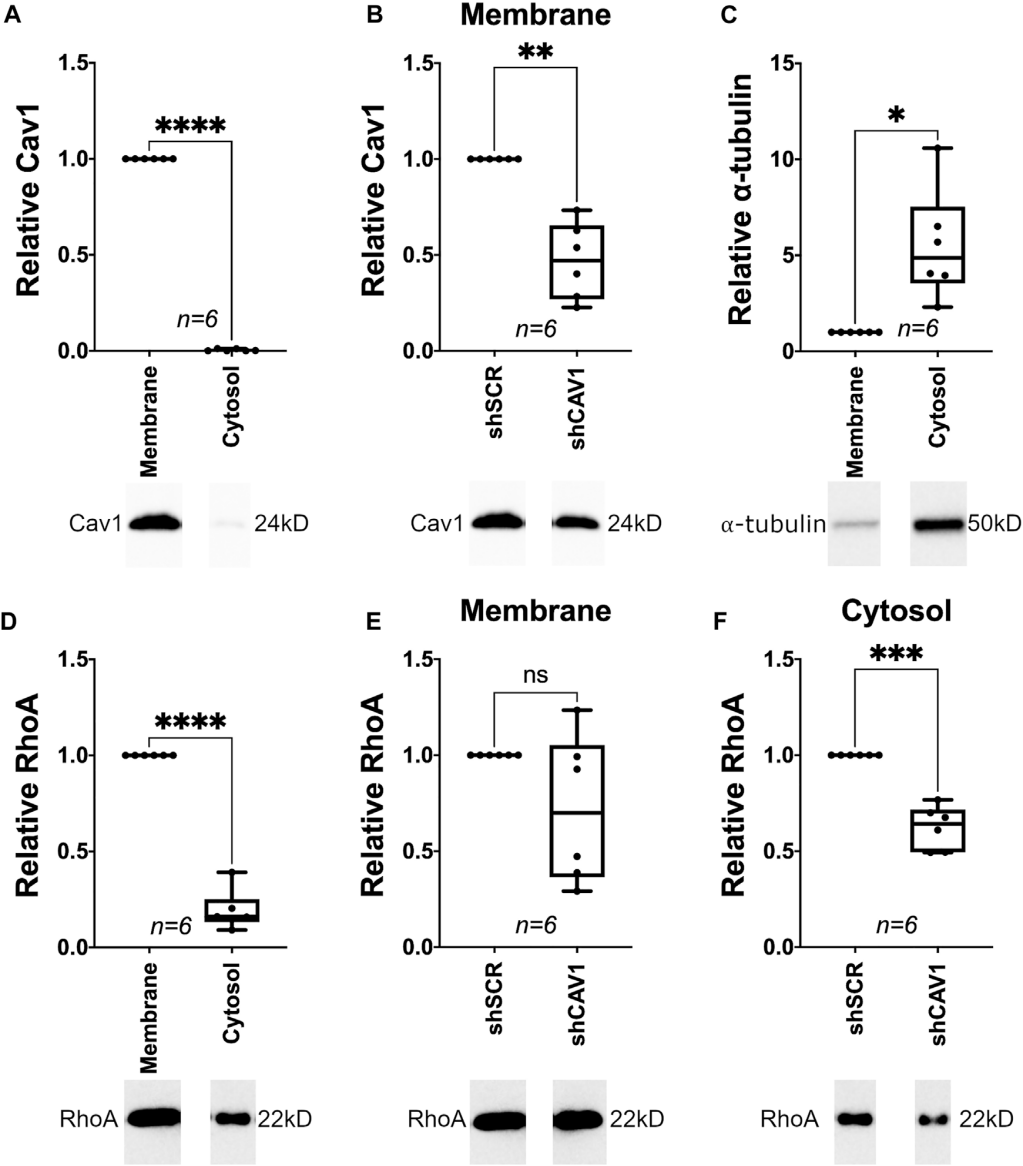
Cellular localization of RhoA in Cav1-competent and Cav1-deficient human TM cells. (**(A)**, Top) Under basal conditions, Cav1 protein localized almost exclusively to membrane fraction in TM cells (250-fold, ****p < 0.0001, paired *t*-test, *n* = 6 experiments using five different TM cell strains). (**(A)**, Bottom) Representative blots from a hTM cell strain depicting Cav1 signal in cytosolic verses membrane fractions, normalized to total protein. (**(B)**, Top) For the membrane fraction only, Cav1 protein levels were significantly decreased by approximately 53% in cells treated with CAV1-silencing shRNA compared to cells treated with scrambled shRNA (**p = 0.001, paired *t*-test, *n* = 6 experiments using five different human TM cell strains). (**(B)**, Bottom) Representative blots from an hTM cell strain depicting membrane-bound Cav1 protein abundance, normalized to total protein. (**(C)**, Top) Under basal conditions, α-tubulin protein localized preferentially to the cytosolic fraction in TM cells (5.5-fold, *p = 0.012, paired *t*-test, *n* = 6 experiments using five different TM cell strains). (**(C)**, Bottom) Representative blots from a hTM cell strain depicting α-tubulin signal in cytosolic verses membrane fractions, normalized to total protein. (**(D)**, Top) Under basal conditions, RhoA protein localized preferentially to the membrane fraction (5-fold) as compared to cytosolic fraction (****p < 0.0001, paired *t*-test, *n*= 6 experiments using five different TM cell strains). (**(D)**, Bottom) Representative blots from a hTM cell strain depicting RhoA signal in cytosolic verses membrane fractions, normalized to total protein. (**(E)**, Top) For the membrane fraction only, RhoA protein levels were not significantly different in cells treated with CAV1-silencing shRNA compared to cells treated with scrambled shRNA (*p* = 0.13, paired *t*-test, *n* = 6 experiments using five different human TM cell strains). (**(E)**, Bottom) Representative blots from a hTM cell strain depicting membrane-bound RhoA protein abundance, normalized to total protein. (**(F)**, Top) For the cytosolic fraction only, RhoA protein levels were significantly decreased by approximately 38% in cells treated with CAV1-silencing shRNA compared to cells treated with scrambled shRNA (***p = 0.0004, paired *t*-test, *n* = 6 experiments using five different human TM cell strains). (**(F)**, Bottom) Representative blots from an hTM cell strain depicting cytosolic RhoA protein abundance, normalized to total protein.

Our data is consistent with previous studies, showing RhoA localizes to both the cytoplasm and plasma membrane (Adamson et al., 1992); translocating from the cytosol to the plasma membrane upon activation (Philips et al., 1993; Boivin and Beliveau 1995; Fleming et al., 1996; Kranenburg et al., 1997; Michaelson et al., 2001). Although, RhoA is negatively regulated when compartmentalized within caveolar lipid rafts (Nuno et al., 2012). Thus, we interrogated the effect of Cav1 expression levels on RhoA localization in TM cells. Under baseline conditions, Cav1 near exclusively localized to the membrane, with close to undetectable levels in the cytosolic fraction of TM cells (Figure 7A, 0.004 ± 0.002, *n* = 6, *p* < 0.0001). Upon knockdown, Cav1 was reduced by 53% (Figure 7B, 0.47 ± 0.08, *n* = 6, *p* = 0.001). As expected, α-tubulin preferentially localized to the cytosolic as compared to membrane cellular compartment (Figure 7C, 5.5 ± 1.2, *n* = 6, *p* = 0.012). Similar to immunofluorescence studies under basal conditions (Figure 6), RhoA localized predominantly in the membrane verses cytosolic fractions (Figure 7D, 0.19 ± 0.04, *n* = 6, *p* < 0.0001). Interestingly, we found that RhoA protein levels in the membrane fraction were not significantly different between Cav1-competent and Cav1-deficient TM cells (Figure 7E, 0.72 ± 0.16, *n* = 6, *p* = 0.13). However, RhoA was significantly reduced in cytosolic fractions by 38% in Cav1-deficient verses Cav1-competent TM cells (Figure 7F, 0.62 ± 0.05, *n* = 6, *p* = 0.0004). These data suggest that while membrane-bound RhoA expression is unchanged, cytoplasmic RhoA expression is downregulated in Cav1-deficient TM cells.

## Discussion

Caveolae in the TM are crucial for homeostatic regulation of IOP and CO resistance (Elliott et al., 2016; De Ieso et al., 2020), however, the biochemical and biomechanical mechanisms underpinning caveolae-mediated regulation of IOP and CO resistance are not well understood. One way the TM participates in the regulation of CO resistance is by modulating contractile tone, where RhoA GTPases plays a central role (Rao and Epstein 2007; Ren et al., 2016). Importantly, RhoA physically interacts with Cav1 in some cell types (Gingras et al., 1998), so we hypothesized that caveolae sequester/inhibit RhoA in the TM, thus regulating downstream signaling involved in contractile tone that is mechanoresponsive. In this way, we found that 24 h of cyclic stretch induced downregulation of Rho/ROCK activity, and that this process was in part Cav1-dependent. Consistent with their functional interactions, we found that RhoA colocalized at the plasma membrane with Cav1, and signaling through MLC was blunted upon treatment with Cav1 scaffolding domain mimetics. Next, we demonstrated that Cav1 negatively regulated Rho GTPase and ROCK activity in TM cells. Taken together, these data support our hypothesis that caveolae sequester/inhibit RhoA *via* interaction with the Cav1 scaffolding domain in the TM to regulate Rho GTPases involved in mechanoresponsive contractile tone. However, more work is needed to confirm direct interaction between RhoA and Cav1 in TM cells.

Work here showed that Cav1 suppression in human TM cells resulted in elevated MLC phosphorylation, which is a surrogate indicator for Rho/ROCK activity and contractile tone (Kimura et al., 1996; Kureishi et al., 1997; Kaneko-Kawano et al., 2012). However, it is possible that other upstream signaling molecules were responsible for elevated MLC phosphorylation in response to Cav1 knockdown. Other pathways upstream of MLC phosphorylation include the Ca^2+^/Calmodulin-dependent myosin light chain kinase pathway (Kamm and Stull 1985; Gallagher et al., 1997) and protein kinase C-mediated pathways (Masuo et al., 1994; Kitazawa et al., 1999). We used a Rho activity assay to confirm that elevation of phosphorylated MLC in response to Cav1 suppression was specifically due to elevated levels of GTP-bound Rho. Thus, we found that Rho activity was increased in Cav1-deficient TM cells where pMLC was elevated, however, this does not exclude the involvement of other aforementioned pathways, especially considering the variety of signaling molecules Cav1 is known to regulate. For example, Cav1 regulates protein kinase C, whereby PKC-driven arterial contraction is increased in Cav1 KO mice (Shakirova et al., 2006). Future work will involve testing whether Cav1 suppression in the TM might also trigger these alternative pathways such as PKC signaling, so to further understand how caveolae regulate contractile tone in the TM.

Our findings are the first to show that Cav1 negatively regulates Rho/ROCK signaling in TM cells. This supports previous findings that Cav1 is a negative regulator of signal transduction in multiple pathways. For example, the Cav1 scaffolding domain (aa 82–101) is implicated in the negative regulation of p42/44 mitogen-activated protein kinase cascade (Engelman et al., 1998), eNOS (Bucci et al., 2000; Gratton et al., 2003; Reina-Torres et al., 2020), TGF-β1/Smad signaling (Lu et al., 2018), protein kinase A (Razani et al., 1999; Razani and Lisanti 2001), vascular endothelial growth factor receptor-2 (Labrecque et al., 2003) and various PKC isoenzymes (Oka et al., 1997). It has been suggested that Cav1 inhibits these signaling molecules by stabilizing them in an inactive conformation within caveolae (Okamoto et al., 1998). Furthermore, Cav1 also negatively regulates Rho activity in certain cell types. For instance et al. (1998) showed that Cav1 physically interacts with RhoA in endothelial cells, where it was proposed to have inhibitory action although this was not empirically demonstrated in this study. Additionally, work from Taggart et al. (2000) revealed that introduction of the Cav1 scaffolding domain peptide to smooth muscle cells isolated from uterine horns of late-term pregnant Sprague–Dawley rats inhibited agonist-induced translocation of RhoA to the plasma membrane, thus resulting in RhoA inhibition. The molecular communication, and negative regulation of RhoA by Cav1 also translates to physiological effectors, whereby smooth muscle contractility is elevated in the absence of Cav1 (Shakirova et al., 2006). Previous evidence of negative regulation of Rho/ROCK activity by Cav1 has also been demonstrated in cells of the outflow pathway. In one study, actin stress fiber formation (an effector of Rho/ROCK activity) was elevated in Cav1-deficient human TM cells (Aga et al., 2014), aligning with findings from this study.

We were surprised to find that cytosolic RhoA expression was suppressed in Cav1-deficient TM cells, despite elevated Rho/ROCK activity. A similar finding was reported where aortic smooth muscle cells extracted from Cav1 knock out mice exhibited reduced RhoA expression compared to wild type, despite elevated smooth muscle contractility and Rho/ROCK activity (Nuno et al., 2012). In this study, researchers also showed that RhoA was negatively regulated when localized within caveolae, and positively regulated when localized within non-caveolar lipid rafts, since depletion of non-caveolar lipid rafts in Cav-1 knock out mice reduced smooth muscle contractility (Nuno et al., 2012). Intriguingly, we found that membrane-bound RhoA expression was unchanged in Cav1-competent verses Cav1-deficient TM cells, despite the fact that RhoA translocates from the cytosol to the plasma membrane upon activation (Philips et al., 1993; Boivin and Beliveau 1995; Fleming et al., 1996; Kranenburg et al., 1997; Michaelson et al., 2001). Thus, we theorize that in the absence of Cav1, RhoA translocates from caveolar to non-caveolar lipid rafts, where RhoA becomes active. Specifically, Cav-1 might stabilize and inhibit RhoA protein thereby preventing its degradation, as previously suggested (Okamoto et al., 1998; Nuno et al., 2012), and it is likely to accomplish this via the Cav1 scaffolding domain. It is important to note that a change in the cytosolic fraction of RhoA does not allow conclusions on the subcellular distribution of active Rho-GTP, and more work is needed to confirm this in future studies.

**FIGURE 8:**
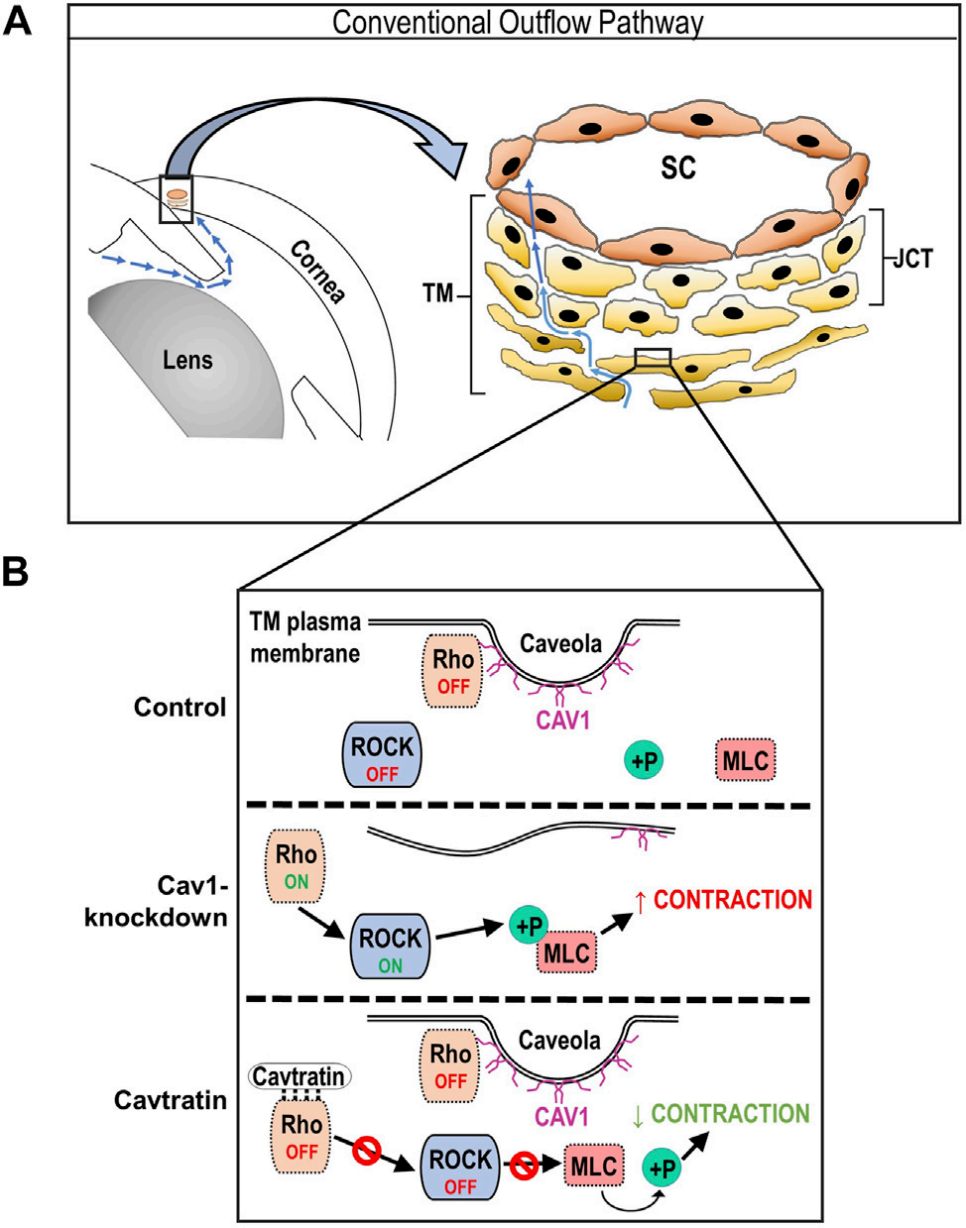
Schematic of hypothesized regulation of Rho/ROCK activity by Cav1 in TM cells. **(A)** Cartoon showing components of the irideocorneal angle, highlighting aqueous humor flow (blue arrows) into and through the conventional outflow pathway. A higher magnification illustration of the conventional outflow pathway, depicting the TM, juxtacanalicular tissue (JCT) and SC on the right. **(B)** Diagram demonstrating results of the present study, showing the status of Rho/ROCK activity and MLC phosphorylation in Cav1 competent, Cav1 deficient, and cavtratin-treated TM cells. For example, we propose that in the presence of Cav1, RhoA localizes to the plasma membrane, where it is sequestered and inhibited via physical interaction with the Cav1 scaffolding domain, leading to a low basal level of Rho/ROCK activity and MLC phosphorylation. In the absence of Cav1, we propose that RhoA is liberated and basal Rho/ROCK activity and MLC phosphorylation is elevated. Following treatment with the Cav1 scaffolding domain peptide, cavtratin, we hypothesize that RhoA is inhibited, leading to reduced ROCK activity and subsequent MLC dephosphorylation.

There is an optimal Cav1 scaffolding domain binding motif in the switch I region of RhoA (Couet et al., 1997), so we hypothesized that binding of RhoA to the Cav1 scaffolding domain prevents its interaction with effector proteins, thus negatively regulating its activity. However, it is important to note that this Cav1 motif is not likely to be the only motif involved with RhoA interaction as RhoB possesses an identical sequence and is not found in caveolae-enriched fractions (Gingras et al., 1998). Nevertheless, we showed that Rho/ROCK signaling is negatively regulated by the Cav1 scaffolding domain. We used two different Cav1 scaffolding domain peptides, cavtratin and cavnoxin to mimic elevation of Cav1 scaffolding domain activity. Cavtratin contains an intact Cav1 scaffolding domain (amino acids 82–101), and cavnoxin is identical to cavtratin, except it contains three inactivating substitutions (T90,91,F92A), disrupting some of the inhibitory actions of the scaffolding domain on eNOS, although some eNOS-modulating activities are likely mediated by the 82–88 segment (Bernatchez et al., 2005). There are two main advantages for using peptides instead of upregulating Cav1 in human TM cells. 1) There is less chance of off target effects associated with the use of viral vectors or transfection reagents. 2) Use of the peptides provides extra insight as to the specific role of the Cav1 scaffolding domain on Rho/ROCK activity. In our study, we found that 24-h treatment with either cavtratin or cavnoxin caused a significant reduction in phosphorylated MLC in human TM cells, without evidence of morphological changes or cytotoxicity (Supplementary Figure S6). These findings align with previous work in smooth muscle cells, demonstrating the inhibitory effect of the Cav1 scaffolding domain on Rho GTPase activity (Taggart et al., 2000). Although TM cells do not express eNOS (Chang et al., 2015; Patel et al., 2020; Reina-Torres et al., 2020; van Zyl et al., 2020), the mechanism by which the Cav1 scaffolding domain negatively regulates RhoA activity in the TM could be mechanistically analogous to the Cav1/eNOS interaction noted in endothelial cells (Garcia-Cardena et al., 1997; Ju et al., 1997). The eNOS protein binds to Cav1 *via* the scaffolding domain (aa 82–101) (Garcia-Cardena et al., 1997; Ju et al., 1997), and it is possible that like eNOS, RhoA also binds to, and is inhibited by, the Cav1 scaffolding domain. We noted that compared to cavtratin, the effect of cavnoxin on MLC dephosphorylation was not as pronounced in human TM cells. This suggests that the F92 residue in the Cav1 scaffolding domain, which is necessary for the inhibitory role of the Cav1 scaffolding domain on eNOS, might also be necessary for the inhibitory role of the Cav1 scaffolding domain on RhoA. Thus, our results are consistent with the idea that like endogenous Cav1, cavtratin sequesters and inhibits RhoA resulting in a reduction in Rho/ROCK signaling, and subsequently a reduction in MLC phosphorylation (Figure 8). However, a limitation of using Cav1 scaffolding domain peptides to test this hypothesis is that the peptides will fail to locate to the same cellular compartments as full length Cav1, thus not behaving exactly like endogenous Cav1. In future work, it will be important to measure how the peptides affect outflow facility in *ex vivo* wild-type and Cav1 KO mouse eyes. We predict that perfusing the peptides will rescue the reduction in outflow facility observed in Cav1 KO eyes.

In contrast to our findings, there is also substantial evidence that Cav1 positively regulates Rho/ROCK activity and contractility in certain cell types (Grande-García et al., 2007; Peng et al., 2007; Joshi et al., 2008; Goetz et al., 2011; Yang et al., 2011; Parton and Del Pozo 2013; Echarri and Del Pozo 2015). Cav1 positively regulates Rho activity in cancer cells (Joshi et al., 2008; Goetz et al., 2011), endothelial cells (Yang et al., 2011), and fibroblasts (Grande-García et al., 2007). In these, cells, Cav1 promotes RhoA activity via indirect inhibition of the endogenous RhoA inhibitor, p190RhoGAP (Grande-García et al., 2007; Goetz et al., 2011). Src kinases activate p190RhoGAP (Arthur et al., 2000; Brouns et al., 2001; Meng et al., 2004), causing a reduction in RhoA signaling. Src kinases also phosphorylate Cav1 at Y14 (Li et al., 1996; Joshi et al., 2008), and phosphorylation of Cav1 at Y14 inhibits further Src activity *via* the recruitment of C-terminal Src kinase (Cao et al., 2002; Radel and Rizzo 2005), resulting in a negative feedback loop. The loss of Cav1 disrupts this negative feedback loop, resulting in elevated Src-p190RhoGAP signaling, and subsequent reduced Rho activity (Grande-García et al., 2007). The differential regulatory role of Cav1 on Rho activity reported in various preparations is not fully understood, but it might be cell-type dependent. For example, negative regulation of Rho activity by Cav1 appears to occur mainly in smooth muscle cells (Taggart et al., 2000; Shakirova et al., 2006; Nuno et al., 2012). As TM cells are hybrid cells, possessing functional properties of multiple cell types including smooth muscle cells (Stamer and Clark 2017), Cav1 might behave differently in smooth muscle and TM cells, as compared to other cell types. One study showed conflicting evidence to this idea, whereby Cav1-silencing in smooth muscle cells resulted in suppression of TGFβ1-induced contractile phenotype markers such as smooth muscle actin (Gosens et al., 2011). While this study shows that Cav1 has an important role in contractile signaling in smooth muscle cells, it only considers TGFβ1-induced contractile phenotypes, and not TGFβ1-independent signaling. The reason why Cav1 differentially regulates Rho activity in various cell types is not yet understood, but it might be partially due to regulatory influence by the extracellular matrix.

The CO tissue participates in mechanotransduction by adapting to fluctuating biomechanical loads, crucial in IOP homeostasis (Overby et al., 2014; Stamer et al., 2015). Mechanotransduction is the ability of a cell to translate a mechanical stimulus, such as stretch or pressure, into a physiological response. Caveolae respond to mechanical perturbations by disassembling and releasing molecules such as eNOS (Yu et al., 2006) and the putative transcriptional regulator, PTRF (Sinha et al., 2011; Nassoy and Lamaze 2012; Parton and Del Pozo 2013). Caveolae mechanotransduction also results in phosphorylation of Cav1, which drives a feedback loop to produce more caveolae components (Joshi et al., 2012). For the first time, we observed phosphorylation of Cav1 in human TM cells following 24 h of cyclic stretch, which suggests that caveolae in the TM respond, and are sensitive to, mechanical perturbations. This agrees with our previous work showing that cyclic mechanical stretch of TM cells results in dissociation of CAVIN1 from Cav1 (Elliott et al., 2016). Additionally, we showed that 24 h of cyclic stretch induces MLC dephosphorylation in human TM cells, and that this process is in part Cav1-dependent. Interestingly, this effect appeared to be time-dependent, as 1 h of cyclic stretch was not enough to induce MLC dephosphorylation in Cav1-competent human TM cells (Supplementary Figure S4). Previous studies have also failed to demonstrate TM relaxation in response to shorter periods of mechanical stimulation. For example, Ramos et al. (2009) showed that 2 h of mechanical strain in the form of cyclic pressure oscillations had no effect on MLC phosphorylation in human TM cells. In addition, Lakk and Krizaj (2021) showed elevated RhoA activation in TM cells exposed to 1 h of cyclic stretch (6% stretch at 0.5 Hz), in a transient receptor potential vanilloid 4 (TRPV4)-dependent manner. Future work will aim to uncover how changes in the duration of mechanical strain might differentially affect mechanotransduction in the TM.

The stretch-induced cellular relaxation is not a commonly reported phenomenon in other cell types. For example, blood vessels are known to respond to increased pressure by constricting, and to decreased pressure by dilating (Davis and Hill 1999). In vascular smooth muscle cells, mechanosensitive ion channels like calcium channels play an important role in mechanotransduction of stretch into a contractile phenotype (Davis et al., 1992a; Davis et al., 1992b). Stretch stimulates Ca^2+^ influx *via* mechanically gated calcium channels leading to contraction, and this process may be regulated by protein kinase C and Cav1 (Laher and Bevan 1989; Zou et al., 1995; Kawamura et al., 2003; Zeidan et al., 2003; Ducret et al., 2010). Cav1 also plays a role in stretch-induced contraction in mesangial cells, whereby 24 h of cyclic stretch induced RhoA activation, and this process was dependent on the integrity of caveolae and on physical association of Cav1 with RhoA (Peng et al., 2007). Although the observed stretch-induced MLC dephosphorylation is uncommon in the context of cardiovascular or renal physiology, it does make sense in the context of the CO pathway. Inhibition of Rho/ROCK signaling causes relaxation of the TM, resulting in reduced IOP and CO resistance (Rao and Epstein 2007; Ren et al., 2016). Therefore, it would make sense that if TM cells “feel” stretch in the form of elevated IOP, they will respond by inhibiting MLC phosphorylation, thereby relaxing, which in turn will increase conventional outflow facility and decrease IOP. Considering the importance of Cav1 and caveolae in the stretch-induced responses noted in vascular smooth muscle and mesangial cells, it would be reasonable to infer that Cav1 might also regulate the stretch-induced response in TM cells, albeit not with the same outcome. Notably, we showed that there was still a trend towards stretch-induced relaxation in Cav1-deficient human TM cells, narrowly approaching significance. This could be due to two possibilities; 1) as Cav1 expression was only partially ablated, it is possible that the remaining caveolae and Cav1 were able to retain partial function in facilitating stretch-induced relaxation. 2) there are other mechanosensitive pathways involved in stretch-induced relaxation of the TM that are independent of Cav1 expression.

Other physiological mechanisms to explain Cav1-facilitated stretch-induced MLC dephosphorylation in TM cells could involve calcium influx through stretch-activated Piezo1 channels, and subsequent activation of large-conductance Ca^2+^-activated K^+^ (BK_Ca_) channels. Piezo1 is expressed in TM cells and is a key transducer of tensile stretch, shear flow and pressure in the TM (Yarishkin et al., 2021). Moreover, Piezo1 can localize in caveolae and stretch-induced Ca^2+^ signaling via Piezo1 is dependent on Cav1 and caveolae integrity in alveolar type II cells (Diem et al., 2020). BK_Ca_ channels regulate TM cell volume and contractility (Wiederholt et al., 2000; Soto et al., 2004) and outflow facility (Soto et al., 2004). BK_Ca_ channels are stimulated by elevation of cytosolic calcium and by depolarization (Wiederholt et al., 2000). Therefore, it would be interesting to see if caveolae facilitate Piezo1 mechanotransduction of stretch signals into calcium influx, subsequently activating BK_Ca_ channels, leading to a reduction in cell contractility. A limitation of this study is that we did not check to see if cyclic stretch induced a reduction in active GTP-bound Rho, which would confirm that stretch-induced MLC dephosphorylation is due to a reduction in Rho/ROCK signaling. It will be important to elucidate which upstream pathways are involved in stretch-induced relaxation, so to better understand the role caveolae play in mechanotransduction in the TM.

In conclusion, we set out to determine whether caveolae regulate mechanotransduction in the TM *via* regulation of the Rho/ROCK pathway, a critical player in the pathogenesis of POAG. Our results provide strong evidence that caveolae are mechanotransducers in the TM, and that caveolae regulate TM contractile tone. These findings identify likely underlying mechanisms linking polymorphisms in the Cav1/2 gene loci with increased risk of POAG.

## Data Availability

The raw data supporting the conclusion of this article will be made available by the authors, without undue reservation.

## Supplementary Material

Supplementary Figure S1 Cav1 knockdown had no effect on αSMA, RhoA, or MLC expression. (**(A)**, Top) Cav1 knockdown had no significant effect on αSMA protein expression in unstretched TM cells (*p* = 0.914, paired t-test, *n* = 8 experiments using 4 different human TM cell strains). (**(A)**, Bottom) Shown is a representative blot from a single experiment displaying αSMA protein abundance, compared to α-tubulin. (**(B)**, Top) Cav1 knockdown had no significant effect on RhoA protein expression in unstretched TM cells (*p* = 0.976, paired t-test, *n* = 7 experiments using 4 different human TM cell strains). (**(B)**, Bottom) Shown is a representative blot from a single experiment displaying RhoA protein abundance, compared to α-tubulin. (**(C)**, Top) Cav1 knockdown had no significant effect on MLC protein expression in unstretched TM cells (*p* = 0.191, paired t-test, *n* = 10 experiments using 6 different human TM cell strains). (**(C)**, Bottom) Shown is a representative blot from a single experiment displaying MLC protein abundance, compared to α-tubulin. Summary data are displayed as box and whisker plots, which show the minimum, 25th percentile, median, 75th percentile, and maximum values of the dataset, plus all individual data points (black circles). Data are expressed as candidate protein expression (corrected for loading as indicated by α-tubulin abundance) in cells treated with shCAV1, normalized to shSCR treated cells.

Supplementary Figure S2 Effect of GTPγS and GDP on GTP-bound Rho. (Top) In control experiments, TM cell lysates were treated with GTPγS or GDP to activate or inactivate Rho, respectively. GDP treatment had no significant effect on levels of GTP-bound (active) Rho (*p* = 0.112, paired t-test, *n* = 8 experiments using 7 different human TM cell strains). GTPγS treatment caused a significant 420% increase in GTP-bound Rho (*****p* < 0.0001, paired t-test, *n* = 8 experiments using 7 different human TM cell strains). (Bottom) Representative blots from a hTM cell strain depicting GTP-bound Rho protein abundance. Untreated (UT) lysate was separated into three vials and treated with GDP, GTPγS, or no treatment (UT). Bands from GTP-bound Rho were all normalized to total Rho protein from UT lysate (input). Total Rho and GTP-bound Rho bands were prepared on separate gels. In some experiments, separate lysates were extracted for each of the UT, GDP, and GTPγS treatments, so each of the GTP-bound Rho bands were normalized to the total Rho protein band from their respective protein lysate sample. Summary data are displayed as box and whisker plots, which show the minimum, 25th percentile, median, 75th percentile, and maximum values of the dataset, plus all individual data points (black circles).

Supplementary Figure S3 Twenty-four hours of cyclic stretch had no effect on expression of Cav1, CAVIN1, αSMA, MLC, or RhoA. **(A)** Cyclic stretch had no significant effect on Cav1 protein levels in Cav1-competent (treated with shSCR) TM cells (*p* = 0.694, paired t-test, *n* = 10 experiments using 6 different human TM cell strains) nor Cav1-deficient (treated with shCAV1) TM cells (*p* = 0.52, paired t-test, *n* = 9 experiments using 5 different human TM cell strains). **(B)** Cyclic stretch had no significant effect on CAVIN1 protein levels in Cav1-competent TM cells (*p* = 0.145, paired t-test, *n* = 7 experiments using 4 different human TM cell strains) nor Cav1-deficient TM cells (*p* = 0.586, paired t-test, *n* = 7 experiments using 4 different human TM cell strains). **(C)** Cyclic stretch had no significant effect on αSMA protein levels in Cav1-competent TM cells (*p* = 0.489 paired t-test, *n* = 8 experiments using 4 different human TM cell strains) nor Cav1-deficient TM cells (*p* = 0.194 paired t-test, *n* = 8 experiments using 4 different human TM cell strains). **(D)** Cyclic stretch had no significant effect on MLC protein levels in Cav1-competent TM cells (*p* = 0.175, paired t-test, *n* = 9 experiments using 5 different human TM cell strains) nor Cav1-deficient TM cells (*p* = 0.353, paired t-test, *n* = 10 experiments using 6 different human TM cell strains). **(E)** Cyclic stretch had no significant effect on RhoA protein levels in Cav1-competent TM cells (*p* = 0.444, paired t-test, *n*=8 experiments using 4 different human TM cell strains) nor Cav1-deficient TM cells (*p* = 0.236, paired t-test, *n* = 8 experiments using 4 different human TM cell strains). Summary data are displayed as box and whisker plots, which show the minimum, 25th percentile, median, 75th percentile, and maximum values of the dataset, plus all individual data points (black circles). Data are expressed as candidate protein expression (corrected for loading as indicated by α-tubulin abundance) in stretched cells, normalized to unstretched cells.

Supplementary Figure S4 One hour of cyclic stretch had no effect on MLC phosphorylation in Cav1-competent TM cells. **(A)** In un-stretched TM cells, Cav1 protein levels were significantly decreased by approximately 35% in cells treated with CAV1-silencing shRNA compared to cells treated with scrambled shRNA (****p* = 0.0007, paired t-test, *n* = 7 experiments using 4 different human TM cell strains). In stretched TM cells, Cav1 protein levels were similarly decreased by 36% in cells treated with CAV1-silencing shRNA compared to cells treated with scrambled shRNA (***p* = 0.004, paired t-test, *n* = 7 experiments using 4 different human TM cell strains). **(B)** One hour of cyclic stretch had no significant effect on Cav1 phosphorylation in Cav1-competent TM cells (*p* = 0.25, paired t-test, *n* = 7 experiments using 4 different human TM cell strains) nor Cav1-deficient TM cells (*p* = 0.77, paired t-test, *n* = 7 experiments using 4 different human TM cell strains). **(C)** One hour of cyclic stretch had no significant effect on MLC phosphorylation in Cav1-competent TM cells (*p* = 0.87, paired t-test, *n* = 7 experiments using 4 different human TM cell strains). However, one hour of cyclic stretch resulted in a 30% reduction of MLC phosphorylation in Cav1-deficient TM cells (**p* = 0.022, paired t-test, *n* = 7 experiments using 4 different human TM cell strains). Summary data are displayed as box and whisker plots, which show the minimum, 25th percentile, median, 75th percentile, and maximum values of the dataset, plus all individual data points (black circles). Data are expressed as candidate protein expression (corrected for loading as indicated by α-tubulin abundance) in stretched cells, normalized to unstretched cells. For A, data are expressed as candidate protein expression normalized to unstretched, shSCR-treated cells

Supplementary Figure S5 RhoA co-localizes with Cav1 in hTM cells. **(A)** Immunofluorescent microscopic images depicting human TM cell line hTM204, stained for DAPI (blue, top left), RhoA (red, top right), and Cav1 (green, bottom left). Merged signals are depicted on bottom right, with dotted lines representing cross-sections used to measure signal intensities and to highlight colocalization of signal, as referred to in panel B. **(B)** Each graph shows the signal intensities (y-axis) of the Cav1 signal (green) and the RhoA signal (red), as a function of distance (*x*-axis). Each graph is numbered and represents the signal intensities of Cav1 and RhoA along respective dotted lines shown in panel A. Points of coincidental signal peaks (peak defined as maximum *Y* value >10 arbitrary units) are representative of Cav1/RhoA co-localization and are highlighted with arrows. **(C)** Immunofluorescent microscopic images depicting human TM cell line hTM210, stained for DAPI (blue, top left), RhoA (red, top right), and Cav1 (green, bottom left). Merged signals are depicted on bottom right, with dotted lines representing cross-sections used to measure signal intensities and to highlight colocalization of signal, as referred to in panel B. **(D)** Each graph shows the signal intensities (*y*-axis) of the Cav1 signal (green) and the RhoA signal (red), as a function of distance (*x*-axis). Each graph is numbered and represents the signal intensities of Cav1 and RhoA along respective dotted lines shown in panel A. Points of coincidental signal peaks (peak defined as maximum *Y* value >10 arbitrary units) are representative of Cav1/RhoA co-localization and are highlighted with arrows.

Supplementary Figure S6 Cav1 scaffolding domain peptides have no effect on TM cell morphology or viability. Bright field microscopic images depicting human TM cell strain, hTM 210, following 24 h treatment with cavtratin (1 μM), cavnoxin (1 μM), and AP control (1 μM).
