# Supplementary figures and images for: A Role of Caveolae in Trabecular Meshwork Mechanosensing and Contractile Tone
Source: Front Cell Dev Biol. 2022 Mar 17;10:855097. doi: 10.3389/fcell.2022.855097 (PMC8969750; doi:10.3389/fcell.2022.855097)

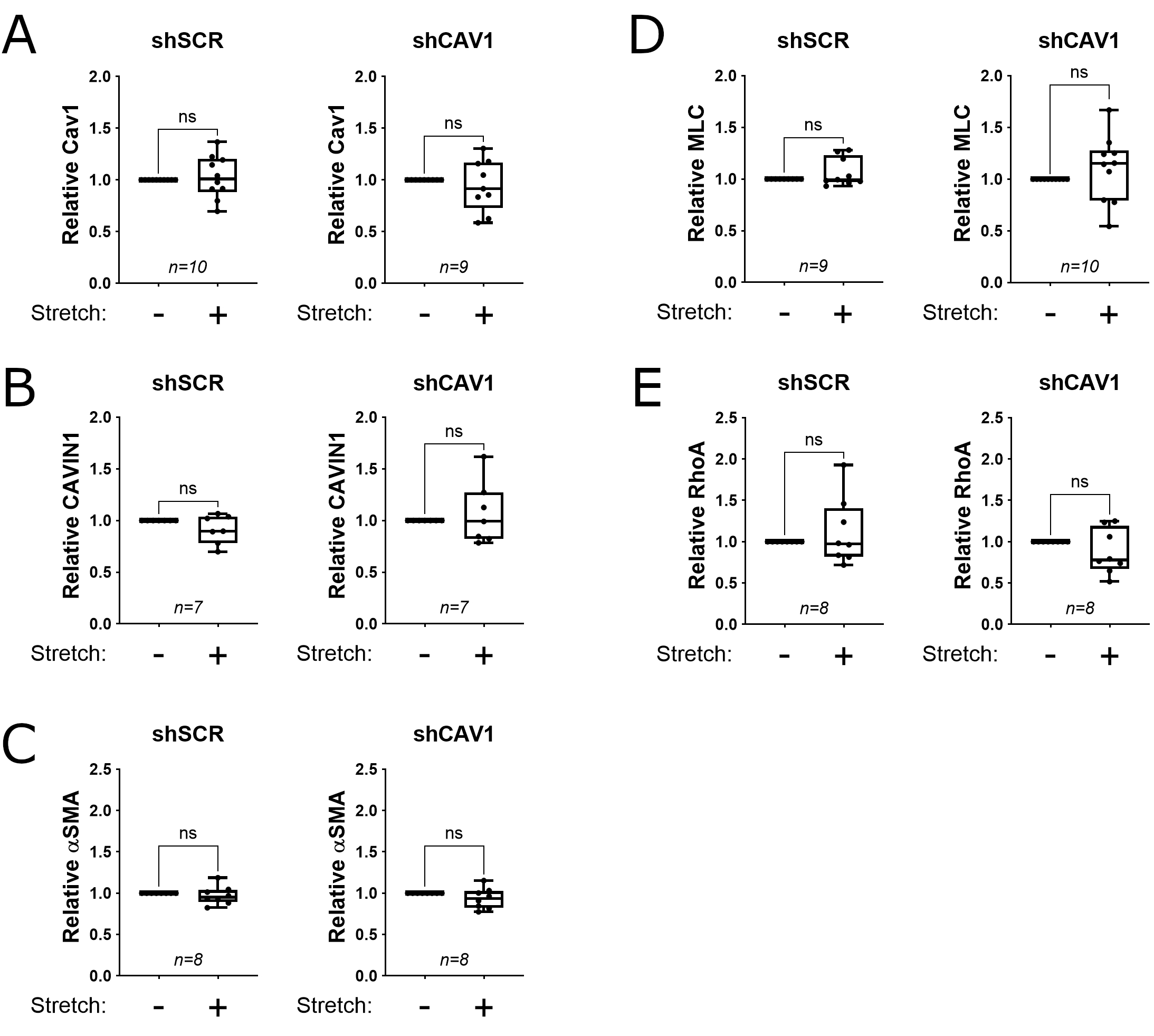

Supplement: Supplementary file 1 [file Image3.JPEG]

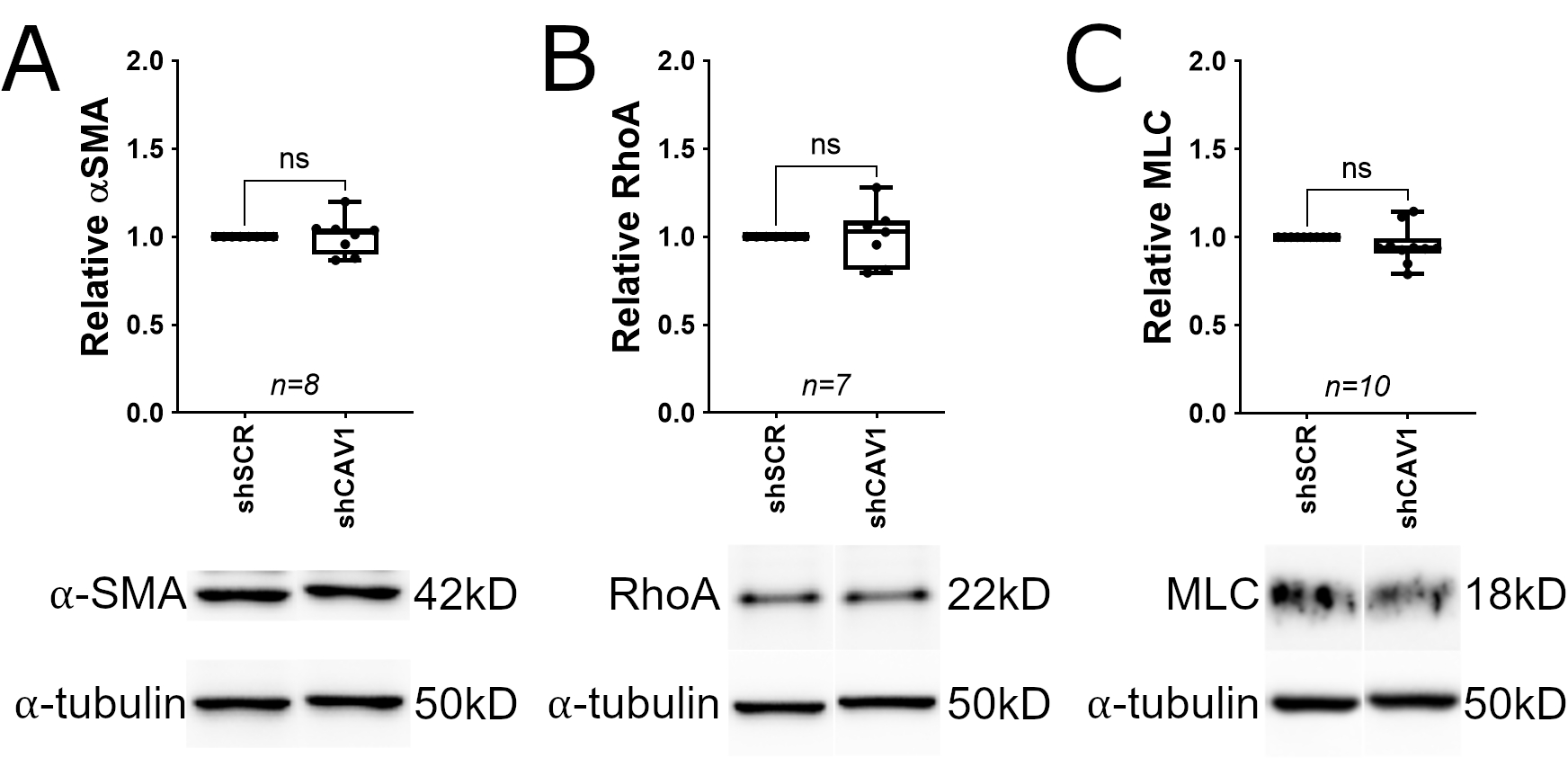

Supplement: Supplementary file 2 [file Image1.JPEG]

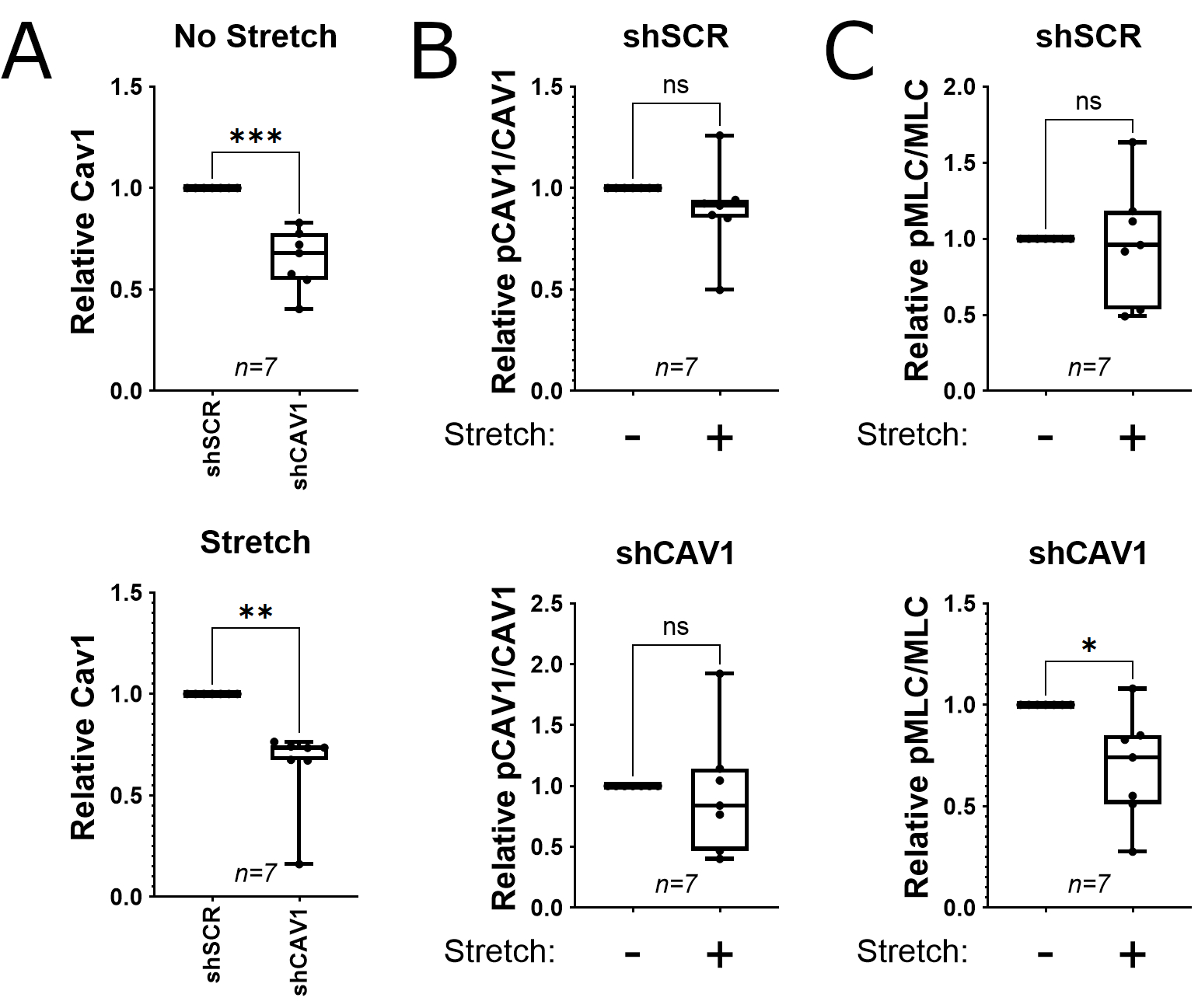

Supplement: Supplementary file 3 [file Image4.JPEG]

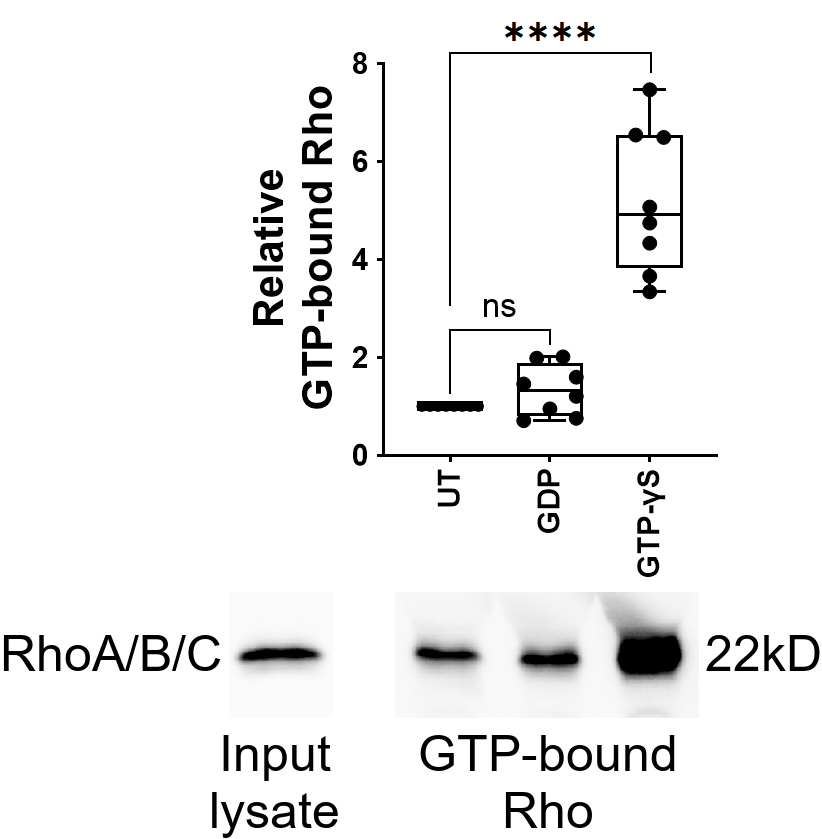

Supplement: Supplementary file 4 [file Image2.JPEG]

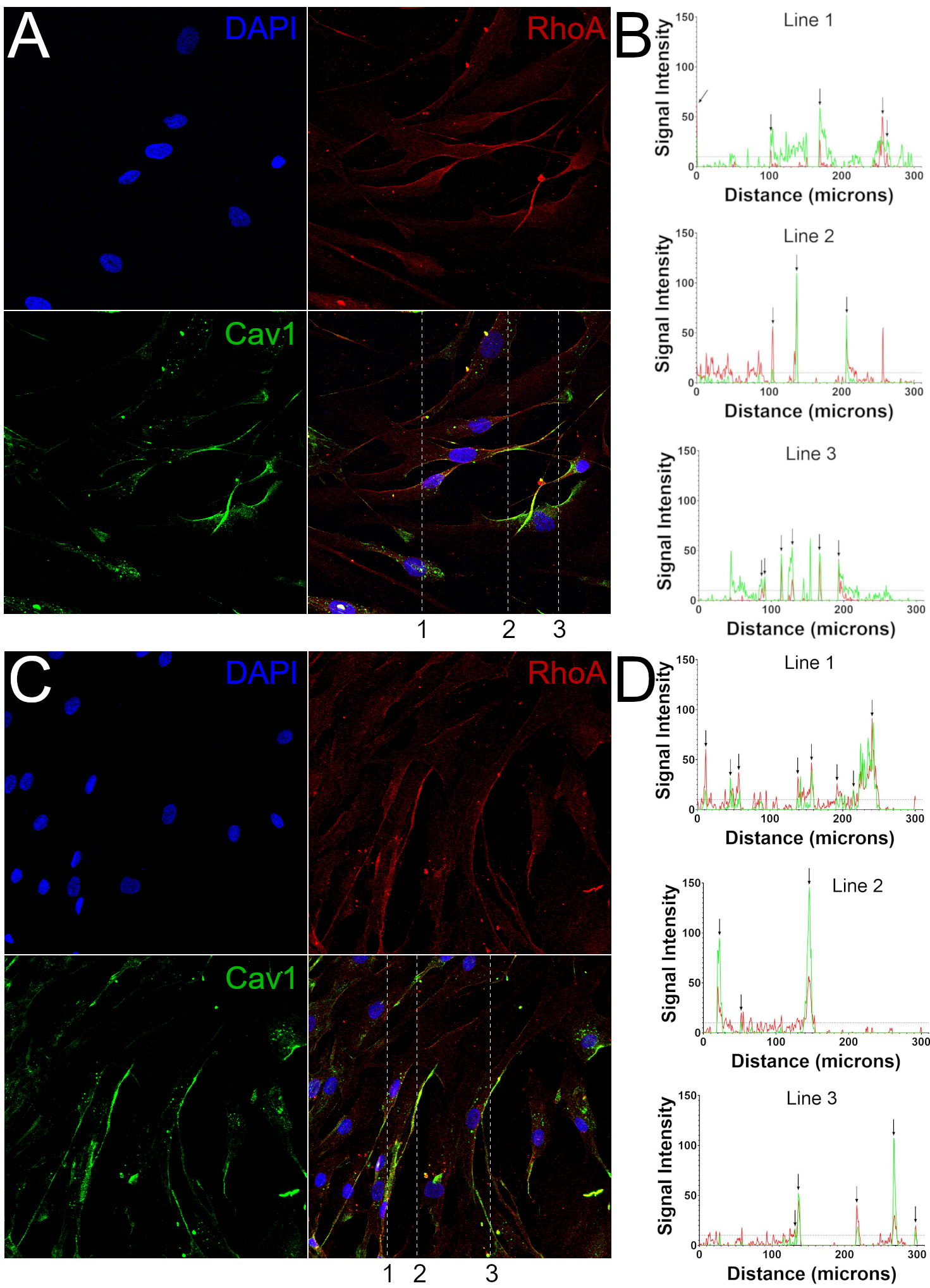

Supplement: Supplementary file 5 [file Image5.JPEG]

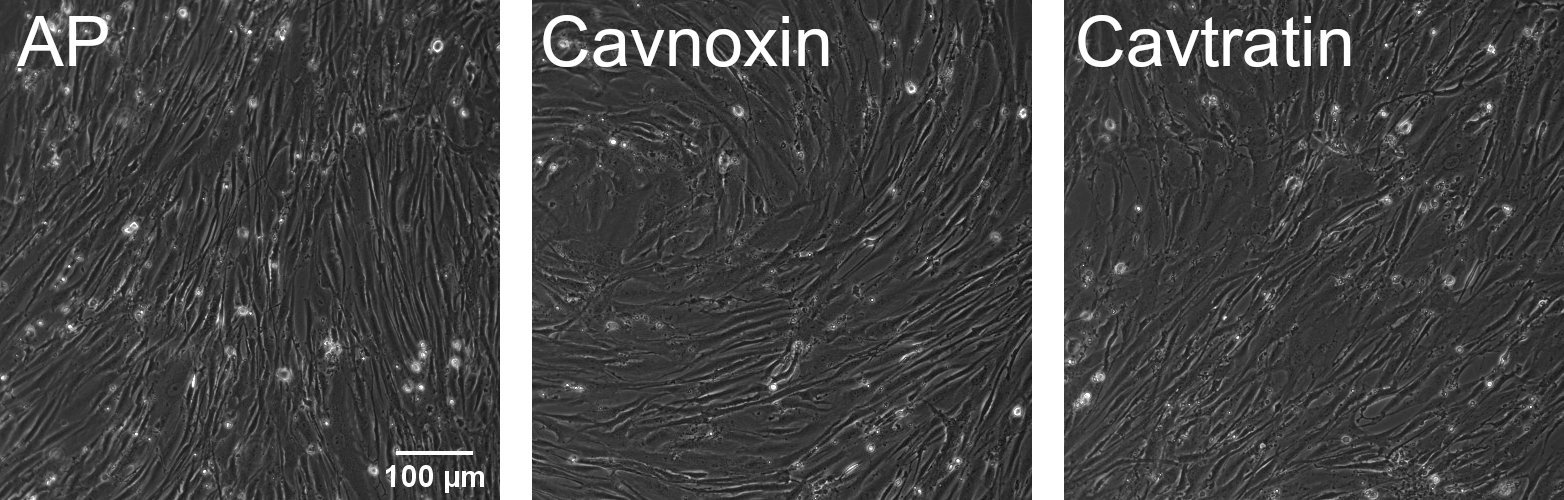

Supplement: Supplementary file 6 [file Image6.JPEG]
